# Infrared Nano‐Spectroscopy of Single Cell Sections at the Ultrastructural Level

**DOI:** 10.1002/nap2.70056

**Published:** 2026-03-24

**Authors:** Florina Marxer, Marta Gallego, Carlos Sanchez‐Cano, Tomasz P. Wrobel, Wolfgang J. Parak, Rainer Hillenbrand, Martin Schnell

**Affiliations:** ^1^ CIC nanoGUNE BRTA Donostia‐San Sebastián Spain; ^2^ Center for Cooperative Research in Biomaterials (CIC biomaGUNE) Basque Research and Technology Alliance (BRTA) San Sebastián Spain; ^3^ Donostia International Physics Center Donostia‐San Sebastian Spain; ^4^ IKERBASQUE Basque Foundation for Science Bilbao Spain; ^5^ Polimero eta Material Aurreratuak: Fisika, Kimika eta Teknologia, Kimika Fakultatea Euskal Herriko Unibertsitatea UPV/EHU Donostia‐San Sebastian Spain; ^6^ Solaris National Synchrotron Radiation Centre Jagiellonian University Krakow Poland; ^7^ Universität Hamburg Hamburg Germany; ^8^ Department of Electricity and Electronics UPV/EHU Leioa Spain

## Abstract

Resin‐embedded cell sections are the standard approach for ultrastructural imaging with electron microscopy. Recently, infrared (IR) nanoimaging has emerged as a label‐free alternative that visualizes cellular structure based on intrinsic molecular contrast. However, assignment of cellular components still requires morphological interpretation. Here, we demonstrate that nano‐FTIR spectroscopy can chemically distinguish subcellular structures by their IR spectral fingerprint. To achieve this, we implemented interleaved referencing to mitigate interferometer drift between sample and reference measurement, reaching a near‐field phase sensitivity of 4 mrad. Combined with an effective medium model to remove resin‐derived IR absorption bands, our approach enables reliable detection of small differences in cellular IR absorption with 50 nm spatial resolution. Using human cervical cancer (HeLa) and mouse osteoblast (MC3T3) cell lines as examples, we spatially resolved intracellular variations in protein and nucleic acid (DNA/RNA)‐associated IR bands. Nucleoli were identified as regions with the highest molecular content within the nucleus, whereas cytoplasm showed cell‐type‐dependent differences: elevated nucleic acid bands in HeLa and reduced in MC3T3 cells. This direct approach for molecular mapping offers opportunities for biomedical research, providing multichannel IR information on individual cell organelles that can be analyzed for understanding cell organization, disease mechanisms, drug delivery and nanoparticle treatments.

## Graphical abstract

Using nano‐FTIR spectroscopy, we resolve subtle differences in infrared absorption among individual subcellular regions of a human cell line with 50 nm spatial resolution. The multichannel information of nano‐FTIR spectra can provide a basis for detailed biochemical interpretation on the ultrastructural level that complement existing electron microscopy observations.

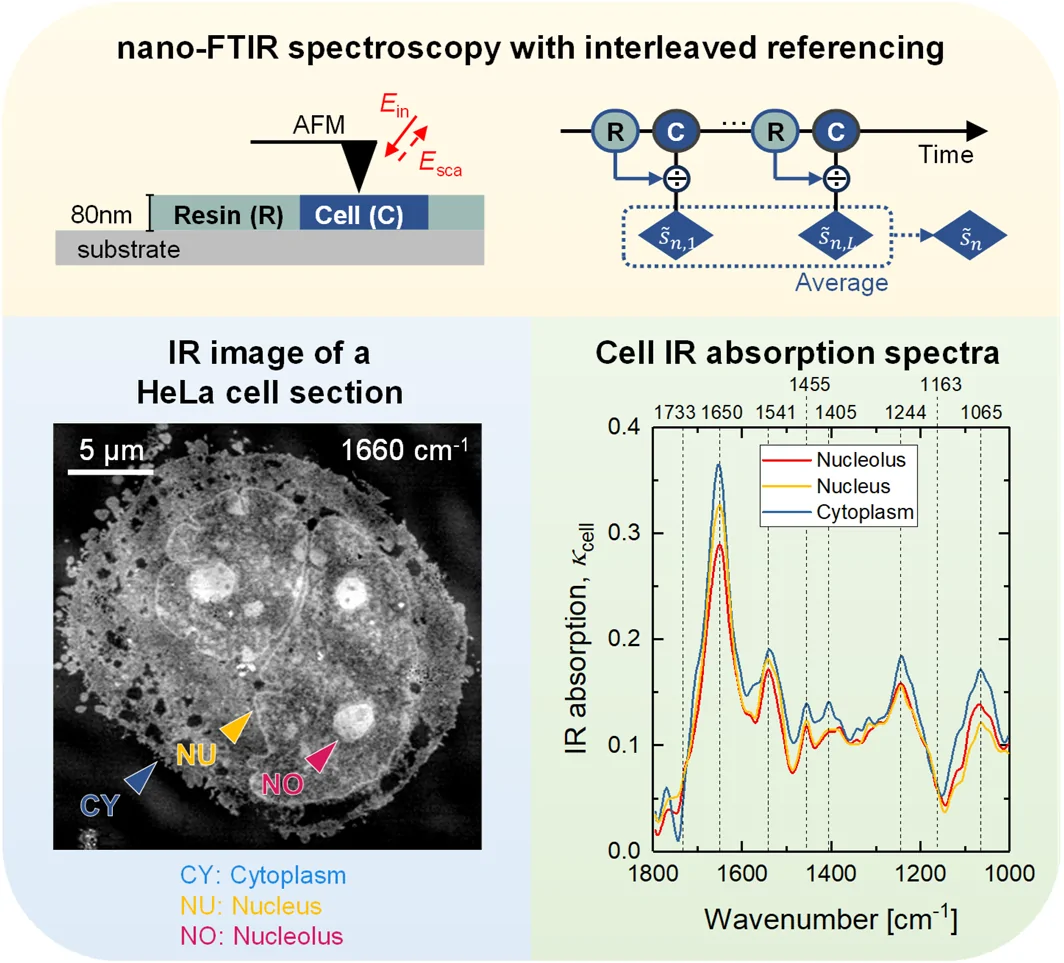

## 1 Introduction

Infrared (IR) spectroscopy enables highly sensitive probing of molecular structure, conformation, interaction, and dynamics in biological systems [1]. When applied to cells and sections of tissue, it probes the vibrational modes of all constituents present in the sample and thus provides a spectroscopic fingerprint of the total chemical composition. This extensive information can be correlated with relevant medical conditions for biodiagnostic purposes. To this end, cell and tissue structures are typically imaged in an IR microspectrometer and the resulting spatio‐spectral datasets are then analyzed on a computer to determine the disease state [2, 3, 4, 5, 6]. Notably, IR spectroscopy is expected to detect biochemical alterations associated with diseases before traditional clinical methods can recognize the related morphological manifestations, potentially leading to new forms of clinical diagnosis [7]. So far, IR spectroscopic investigations are typically limited to the cellular level because diffraction limits the spatial resolution to the micrometer scale. Thus, IR spectroscopy is inadequate for the direct measurement of IR spectral changes within cells unless indirect methods, such as cell fractionation, are applied [8]. In contrast, electron microscopy is routinely applied for the direct investigation of cellular ultrastructure, providing exceptional insights into cell functions and disease‐induced aberrations in individual cell organelles [9]. However, electron microscopy requires staining to visualize structure and consequently the chemical composition of subcellular organelles is typically limited. It is thus desirable to combine the specificity of IR spectroscopy with the spatial resolution of electron microscopy for a most comprehensive analysis of cellular ultrastructure.

Probe‐based nano‐IR techniques overcome the diffraction limit and enable IR imaging and spectroscopy with nanoscale spatial resolution. One promising method is scattering‐type scanning near‐field optical microscopy (s‐SNOM), which has already proven highly successful for the nanoscale chemical identification [10] of protein [11, 12, 13, 14, 15, 16], polymer [17], biological [18], and other organic and inorganic samples [19, 20, 21, 22, 23]. s‐SNOM uses the sharp metal tip of an atomic force microscope (AFM) to focus IR light to strongly confined near fields around the tip apex. By bringing the tip in close proximity to a sample surface, the tip couples with the sample via the near field and the local optical properties of the sample can be measured with nanoscale spatial resolution. An alternative method for nano‐IR is atomic force microscopy infrared (AFM‐IR) spectroscopy, which uses the AFM tip to locally detect IR absorption in the sample based on the photothermal expansion effect [24, 25].

Both s‐SNOM and AFM‐IR hold potential for the label‐free, chemical mapping of cellular ultrastructure. So far, prior efforts on nano‐IR imaging of cells can be divided into two main approaches. One approach focuses on the investigation of whole cells [26, 27] to analyze disease [28, 29, 30] and biological processes [31, 32], to localize drugs uptake [33, 34], to map optical properties [35], and imaging of live cells [36]. The other approach focuses on the imaging of thin cell sections instead: cells are resin‐embedded and cut to a thin section with an ultramicrotome, exposing the inner cellular ultrastructure for direct IR nano‐imaging. The resulting IR images reveal the cell section’s morphological features and enable identification of subcellular structures based on their shape and spatial context, in good alignment with established protocols for ultrastructural pathology and providing this advantage over bulk cell samples [37, 38, 39, 40].

So far, nano‐IR studies of cell sections have primarily focused on imaging at one or few selected frequencies. Chemical information was thus limited and nano‐IR imaging was used mainly to highlight morphological features. Nano‐FTIR spectroscopy—a derivative of s‐SNOM—can, in principle, record complete IR fingerprint spectra from subcellular regions [37], thereby enabling their identification based on IR spectral features rather than relying on morphological interpretation. However, several important aspects require clarification to establish nano‐FTIR spectroscopy as a tool for cellular analysis: (i) nano‐FTIR spectroscopy should be able to resolve both strong and weak cellular IR absorption lines with sufficient signal to noise ratio and spectral resolution, (ii) it should be capable of detecting subtle changes in the peak height of these IR absorption lines that may occur as a result of a change in chemical composition, and (iii) the influence of the resin material on the nano‐FTIR spectra needs to be clarified, and particularly how cellular IR absorption can be extracted from the nano‐FTIR spectral data and correlated with conventional (far‐field) IR spectroscopy in terms of peak height and peak position.

Here, we present a method for high‐sensitivity nano‐FTIR spectroscopy of resin‐embedded cell cuts. We first developed interleaved referencing that eliminates the unavoidable interferometer drift and achieves an unprecedented near‐field phase sensitivity of 4 mrad (∼0.2°) across the IR fingerprint spectral band. We further applied an effective medium model to remove the IR absorption bands from the resin material and extract the IR absorption spectra of the cell material. Together, these two techniques allow to resolve cellular vibration bands in the IR fingerprint spectral region at a spatial resolution of 50 nm and detect small spectral differences across different regions of cell sections. We demonstrate the method with the example of two well‐studied cell lines—a human cervical cancer cell line (HeLa) and a mouse osteoblast cell line (MC3T3)—and document the IR spectral contrast of distinct subcellular organelles with a focus on the protein and nucleic acid content.

## 2 Results

### 2.1 Interleaved Referencing for Nano‐FTIR Spectroscopy

Figure 1a shows the nano‐FTIR spectroscopy setup employed in this study (neaSNOM, Attocube Systems GmbH). A broadband mid‐IR continuum laser based on the difference frequency generation is used as a light source. The laser beam, $E_{\text{in}}$, is focused with a parabolic mirror PM onto the metallic tip (PPP‐NCSTAu and Nanoworld) of an atomic force microscope AFM. The tip‐scattered field, $E_{\text{sca}}$, is collected with a parabolic mirror PM and analyzed with an asymmetric Fourier transform interferometer comprising beam splitter BS, a wire‐grid attenuator ATT, a translatable mirror M, and a mercury‐cadmium‐telluride detector DET [17]. To remove unwanted background scattering from the tip shaft and sample, the AFM tip is vibrated vertically at a frequency, Ω=154kHz, and with an amplitude of 107 nm, and the detector signal, $I_{\det}$, is demodulated at a higher harmonic n of the tip vibration frequency, nΩ. Nano‐FTIR spectroscopy is performed by (i) positioning the AFM tip on a region of interest (ROI) within the cell, (ii) recording the demodulated detector signal as a function of the reference mirror position, δ, to obtain a near‐field interferogram, $I_{n}\left(\delta \right)$, and (iii) performing a Fourier transform of $I_{n}\left(\delta \right)$ to obtain a near‐field spectrum, ${\tilde{S}}_{n}\left(\omega \right)$. Typically, this near‐field spectrum, ${\tilde{S}}_{n}\left(\omega \right)$, is divided by the spectrum taken at a well‐defined reference on the sample, ${\tilde{S}}_{n}^{R}\left(\omega \right)$,—in this study, this is the resin material adjacent to the cell—to eliminate the instrument’s response function and spectral characteristics of the source. The result is a referenced near‐field spectrum from the ROI [41],

(1) $${\tilde{s}}_{n}\left(\omega \right)=s_{n}\left(\omega \right)e^{i\varphi_{n}\left(\omega \right)}={\tilde{S}}_{n}\left(\omega \right)/{\tilde{S}}_{n}^{R}\left(\omega \right),$$

where $s_{n}\left(\omega \right)$ and $\varphi_{n}\left(\omega \right)$ are the referenced near‐field amplitude and phase spectra, respectively, and the tilde indicates a complex‐valued quantity. In case of molecular vibrations, the near‐field amplitude spectrum, $s_{n}\left(\omega \right)$, yields dispersive‐like spectral line shapes similar to far‐field reflection spectroscopy, whereas the near‐field phase spectrum, $\varphi_{n}\left(\omega \right)$, yields absorption‐like spectral lines shapes of the probed molecular vibrations similar to far‐field transmission spectroscopy, allowing for chemical identification on the nanoscale [11, 14, 17, 42]. To suppress the unavoidable interferometer drift during long measurement times, we developed an interleaved referencing approach for nano‐FTIR spectroscopy (Figure 1b). Briefly, instead of acquiring single sample and reference near‐field spectra at long measurement times (Equation 1), a large number (l=1…L) of sample and reference spectra, ${\tilde{S}}_{n,l}\left(\omega \right)$ and ${\tilde{S}}_{n,l}^{R}\left(\omega \right)$, is recorded in quick succession at short measurement times (typically on the order of 10 s), which allows for tracking interferometer drift. The individual sample near‐field spectra, ${\tilde{S}}_{n,l}\left(\omega \right)$, are then divided by the average of the previous and following reference near‐field spectrum, $\left[{\tilde{S}}_{n,l}^{R}\left(\omega \right)+{\tilde{S}}_{n,l+1}^{R}\left(\omega \right)\right]/2,$ to yield an individual referenced near‐field spectrum,

(2) $${\tilde{s}}_{n,l}\left(\omega \right)=\frac{{\tilde{S}}_{n,l}\left(\omega \right)}{\left[{\tilde{S}}_{n,l}^{R}\left(\omega \right)+{\tilde{S}}_{n,l+1}^{R}\left(\omega \right)\right]/2},$$

**FIGURE 1:**
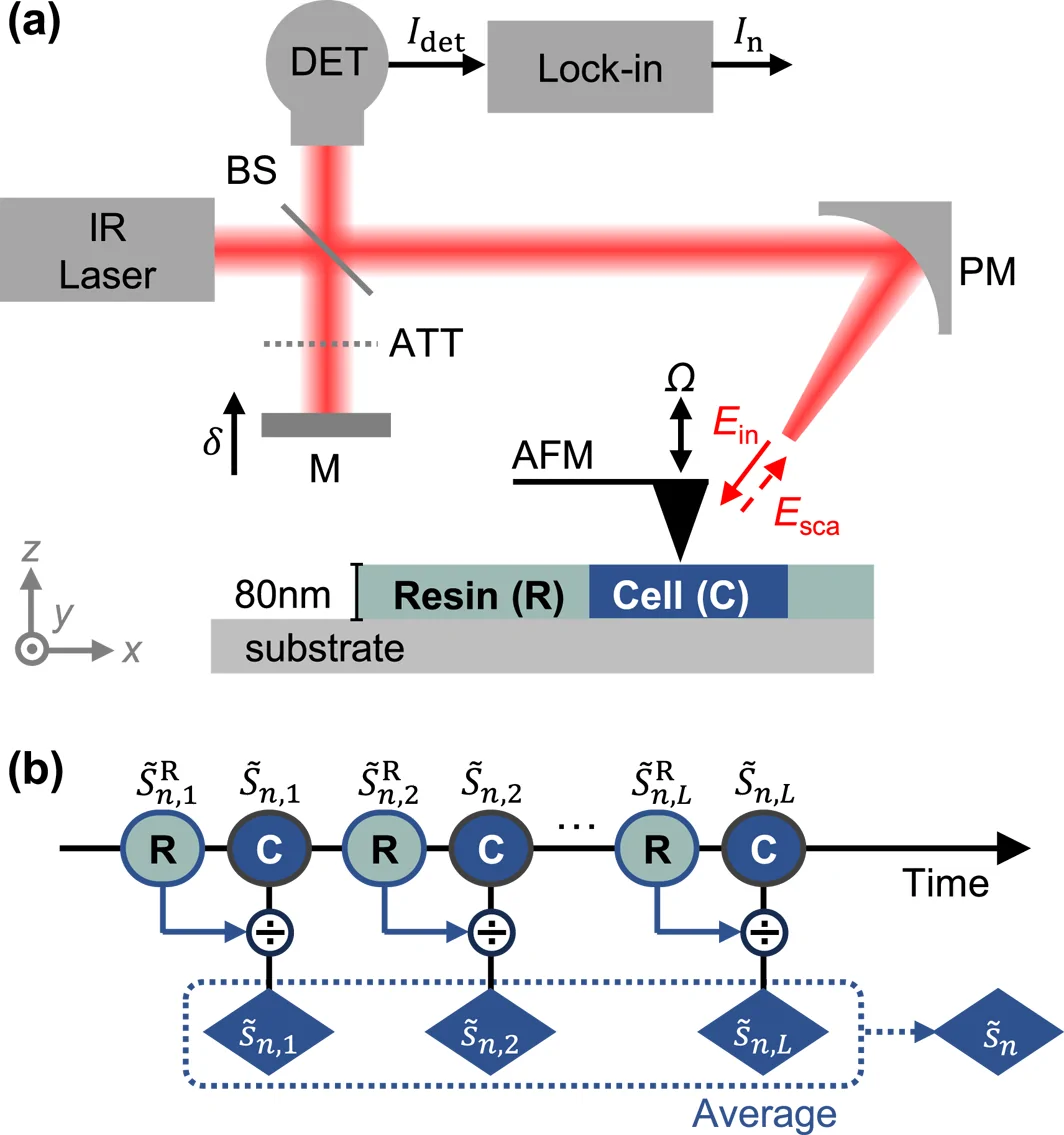
(a) nano‐FTIR spectroscopy of resin‐embedded cell sections. Symbols see text. (b) Simplified schematic of interleaved referencing for nano‐FTIR spectroscopy based on the rapid alternating between reference (R) and sample (C) measurements, ${\tilde{S}}_{n,l}^{R}\left(\omega \right)$ and ${\tilde{S}}_{n,l}\left(\omega \right)$. Signal averaging is performed over the individual referenced spectra, ${\tilde{s}}_{n,l}\left(\omega \right)$.

Signal averaging is performed over the *referenced* near‐field spectra, ${\tilde{s}}_{n,l}\left(\omega \right)$ (Equation 2), to yield the final near‐field spectrum,

(3) $${\tilde{s}}_{n}\left(\omega \right)=s_{n}\left(\omega \right)e^{i\varphi_{n}\left(\omega \right)}=\sum_{l}{\tilde{s}}_{n,l}\left(\omega \right).$$

Interleaved referencing provides the following advantages: (i) it eliminates the need for baseline corrections of near‐field phase spectra and (ii) it enables consistent measurement of the molecular vibration peaks even for long measurement times (see Supporting Information S1: Note S1). As a result, we achieved an unprecedented sensitivity in the near‐field phase of a 4mrad (≈0.2°) across a broad spectral range from 1000 to 1700 cm^−1^ and at a high spectral resolution of $12.5\;{\text{cm}}^{-1}$ (Supporting Information S1: Note S2). This level of sensitivity corresponds to a detection limit of 670 carbonyl bonds, based on literature estimates (assuming 500 carbonyl bonds per 0.15° for second harmonic detection, n=2, see SI Note 6 in ref. [16]). Previous studies reached comparable sensitivity levels, but they were typically limited to a narrower spectral range (∼150–$300\;{\text{cm}}^{-1}$) [14, 16, 21]. With interleaved referencing, the bandwidth is significantly expanded to $700\;{\text{cm}}^{-1}$, making it possible to acquire high‐quality IR fingerprint spectra from nanoscale cell volumes as will be shown next.

### 2.2 IR Nano‐Imaging of Cervical Cancer Cell Sections

Cultured human cervical cancer cells (HeLa) were prepared following a standard protocol for electron microscopy (EM), sectioned to slices of 80 nm nominal thickness and placed on a Si substrate (Methods). We first performed s‐SNOM microscopy to obtain an IR image of the cell section and identify the cellular ultrastructure. To this end, a wavelength‐tunable quantum cascade laser (QCL) was used as a light source and a pseudoheterodyne interferometer for light detection [43]. Figure 2b shows the near‐field phase image of a single cell section that was acquired at the Amide I band (1660 ${\text{cm}}^{-1}$), yielding a map of the protein content within the cell. The cellular ultrastructure was accurately revealed as it can be recognized by comparison with the EM image taken from the same cell section (Figure 2a). Specifically, the nucleoli (NO) appeared with a large near‐field phase signal, indicating their high protein content. The nucleus membrane (NM) is clearly recognizable as a fine line (NM, Figure 2b) as well as other intricate details such as the granular pattern inside the nucleus and the inner structure of nucleoli (digital zooms in Figure 2c). These observations align with the well‐established notion that the nucleus of a cell is a highly nonhomogeneous entity, characterized by distinct substructures that are functionally specialized and linked to various biological processes [44, 45, 46, 47]. These ultrastructural details can be revealed due to the high spatial resolution of s‐SNOM, which in this study was estimated at 48 nm and thus approximately 60 times better than conventional (far‐field) IR microspectroscopy (Supporting Information S1: Note S3). In the nano‐FTIR spectroscopy section below, we will focus on three distinct subcellular regions as marked by color in Figure 2a: nucleus (NU, yellow), nucleolus (NO, red color), and the cytoplasm (CY, blue) adjacent to the nucleus. To this end, near‐field spectra were taken on five selected ROIs on each of the three subcellular regions as indicated by the markers in Figure 2d, to resolve molecular vibrations in the full IR fingerprint spectral range.

**FIGURE 2:**
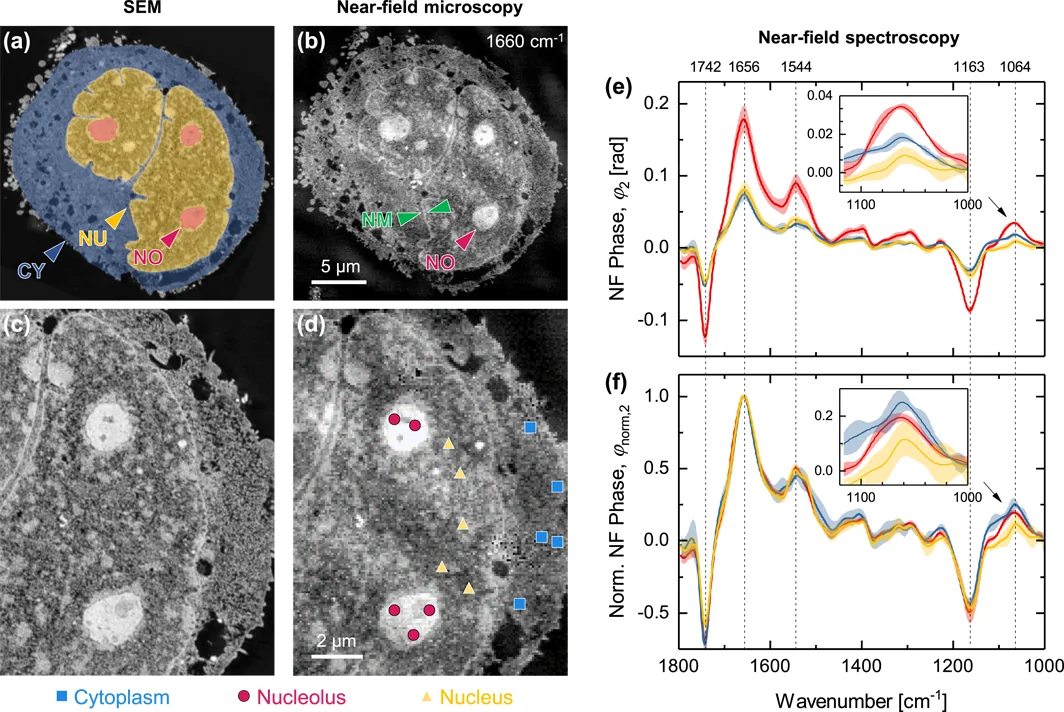
(a) SEM image of a single HeLa cell section. Cytoplasm, nucleolus, and nucleus regions are colored coded as shown in the legend below. (b) Near‐field phase image, $\varphi_{3}$, measured at 1660 ${\text{cm}}^{-1}$ of the same cell section. CY, cytoplasm; NM, nuclear membrane; NO, nucleolus; and NU, nucleus. (c), (d) Zooms in (a) and (b), respectively. (e) Near‐field phase spectra, $\varphi_{2}\left(\omega \right)$, taken at the points indicated in panel (d) and subsequently averaged by class. The solid line shows the class mean spectrum, the transparent band shows the class standard deviation (quantified in Supporting Information S1: Table S2). (f) Near‐field phase spectra, $\varphi_{\text{norm},2}\left(\omega \right)$, normalized to the Amide I peak at 1656 ${\text{cm}}^{-1}$.

### 2.3 Nano‐FTIR Spectroscopy at the Subcellular Level

For a first qualitative interpretation of our data, we analyzed the near‐field phase spectra, $\varphi_{n}\left(\omega \right)$, directly. For samples with small positive permittivity such as the cell sections considered here, the far‐field IR absorbance, A(ω), is approximately proportional to the near‐field phase, $\varphi_{n}\left(\omega \right)$ [41, 42],

(4) $$A\left(\omega \right)\propto \varphi_{n}\left(\omega \right)\;.$$

Figure 2e shows the average near‐field spectra for nucleus (yellow), nucleoli (red), and cytoplasm (blue), $\varphi_{2}\left(\omega \right)$, as obtained by calculating the mean spectrum from the individual near‐field spectra of the same class. The general IR spectral features expected for HeLa cells can already be recognized. Common features to all three subcellular regions are the Amide I (C=O stretching vibration of the protein backbone) at 1656 ${\text{cm}}^{-1}$ and Amide II (N–H bending coupled to C–N stretching vibration of the protein backbone) at 1543 ${\text{cm}}^{-1}$ bands. These bands indicate the high protein content inside the cell as expected [48, 49]. Interestingly, the vibrational bands observed with the nucleolus (red) are 2.2‐fold stronger compared to those of the nucleus (yellow), which indicates a higher material concentration in the nucleolus. Following standard practice from IR spectroscopic cell investigations, we remove this dependency from the material concentration by normalizing the near‐field phase spectra, $\varphi_{2}\left(\omega \right)$, from Figure 2e to the value at the Amide I band,

(5) $$A_{\text{norm}}\left(\omega \right)=\frac{A\left(\omega \right)}{A\left(1656\;{\text{cm}}^{-1}\right)}\propto \varphi_{\text{norm},n}\left(\omega \right)=\frac{\varphi_{n}\left(\omega \right)}{\varphi_{n}\left(1656\;{\text{cm}}^{-1}\right)},$$

where IR sample absorption is expressed relative to the local protein content. With $\varphi_{\text{norm},2}\left(\omega \right)$ (Figure 2f), we observe that the ratio between the Amide I and II bands is about 2:1. Further, all three subcellular regions show a peak at about 1064 cm^−1^, which can be assigned to the $\nu_{s}PO_{2}^{-}$ band (symmetric phosphate stretching vibration) and the C–O stretch of the ribose moiety, present both in DNA and RNA. Although two distinct bands at 1087 and 1052 ${\text{cm}}^{-1}$ are expected from conventional FTIR studies of DNA/RNA, a broad band with a center at 1064 ${\text{cm}}^{-1}$ is observed in our data. We attribute this feature to a possible dehydration‐induced shift to an A‐DNA or Z‐DNA conformation [50] resulting from sample preparation. Although instrument noise can in principle affect the peak shape in interpolated near‐field spectra, this explanation is unlikely as a similar peak shape is observed across all subcellular regions (Figure 2f). On the other hand, the vibration bands in the 1500–1200 ${\text{cm}}^{-1}$ spectral region are less clearly resolved. Specifically, a near zero signal is observed in that spectral range, $\varphi_{\text{norm},2}\left(\omega \right)\approx 0$ and the peak structure appears undefined and wave‐like. Another striking observation is the presence of dips (negative peaks) at 1742 and 1163 ${\text{cm}}^{-1}$, a feature typically not seen in nano‐FTIR spectroscopy. These dips are a result of signal referencing (Equation 2) to the resin material, which also exhibits pronounced IR absorption lines (discussed below).

**FIGURE 3:**
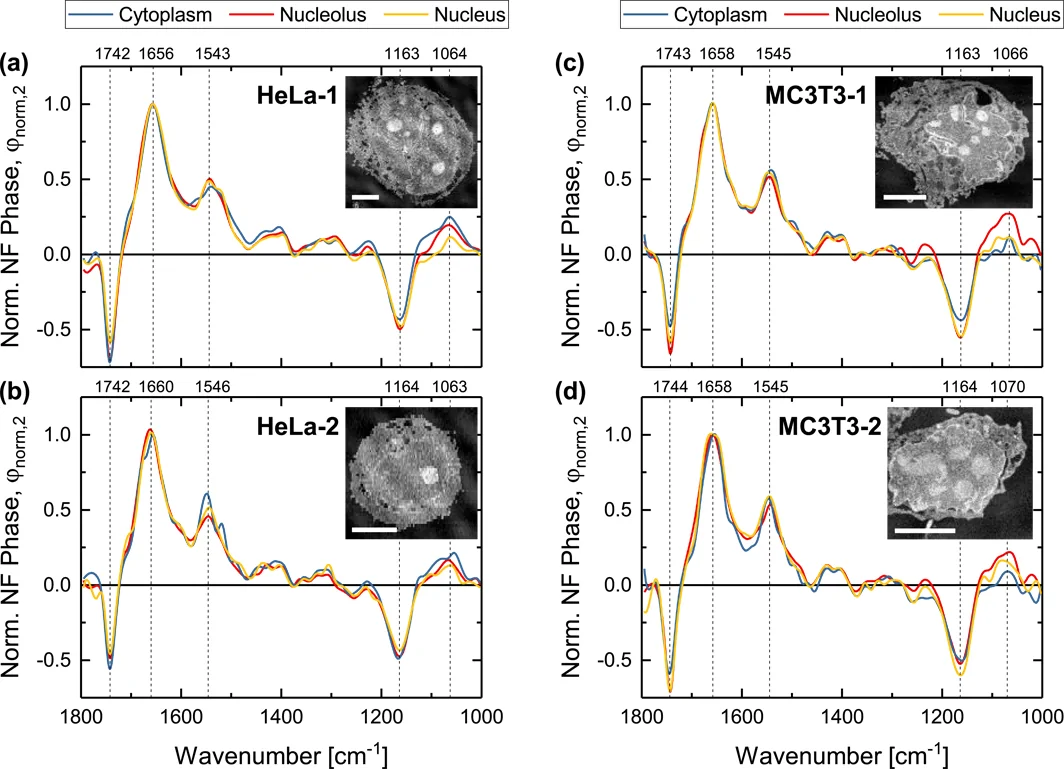
Normalized near‐field phase spectra, $\varphi_{\text{norm},2}\left(\omega \right)$, of two HeLa and two MC3T3 cells are shown in (a–d). Insets show the near‐field phase images, $\varphi_{3}$, of the cells taken at the Amide I band ($1660\;{\text{cm}}^{-1}$). The scale bar is 5μm. Location of ROIs are shown in Supporting Information S1: Figures S6–S9.

Nevertheless, the near‐field phase spectra already allow a qualitative interpretation of chemical contrast. Briefly, $\varphi_{\text{norm},2}\left(\omega \right)$ reveals that all three subcellular regions exhibit largely similar IR absorption signal in the spectral region between 1800 and $1000\;{\text{cm}}^{-1}$, which is a striking result giving their different biochemical composition. The only notable difference appears at the P–O vibration band at $1064\;{\text{cm}}^{-1}$, where the cytoplasm (blue line in Figure 2f) shows slightly increased peak intensity compared to the nucleus (yellow line). This finding is consistent with prior conventional FTIR spectroscopic investigations at the subcellular level, which also reported only minimal spectral variations among subcellular classes. Specifically, although the nucleus is the primary repository of DNA, its IR spectral signature does not show pronounced P–O bands, but instead closely resembles the spectral signature of the cytoplasm [8]. Importantly, we find that the nucleus itself is not homogeneous as it is evidenced by the slightly higher P–O band intensity of the nucleolus compared to the rest of nucleus (Figure 2f).

To test the consistency of nano‐FTIR spectroscopy for ultrastructural investigations, we performed nano‐FTIR spectroscopy on a second HeLa cell. Both HeLa cells show a similar IR absorption in both shape and magnitude (cf. Figure 3a,b). We observe the same small but consistent differences between the different subcellular regions in the P–O stretch regions at around $1064\;{\text{cm}}^{-1}$, which indicates that the presented method provides sufficient sensitivity and reliability for biochemical measurements within cells. Further, we characterized two MC3T3 cells, a mouse osteoblast precursor cell line. Although the MC3T3 cells show a similar IR spectral shape compared to the HeLa cells, there is a marked difference in the intensity of the symmetric P–O band at $1064\;{\text{cm}}^{-1}$, with cytoplasm showing a reduced intensity compared to the nucleus and nucleolus in case of the MC3T3 cells (Figure 3c,d) compared to the HeLa cells (Figure 3a,b).

### 2.4 Extraction of Cell IR Absorption Using Modeling

Quantitative interpretation of near‐field spectra of resin‐embedded cell sections requires addressing two challenges. First, the resin‐derived IR absorption lines must be removed from the near‐field spectra to enable clear identification of the cell‐specific IR absorption bands. Second, the near‐field interaction between the tip and the sample must be described to accurately determine the peak position of the IR absorption bands [41, 51, 52]. The method presented below resolves both challenges by extracting the absorption index spectrum of the cell material (Figure 4a).

**FIGURE 4:**
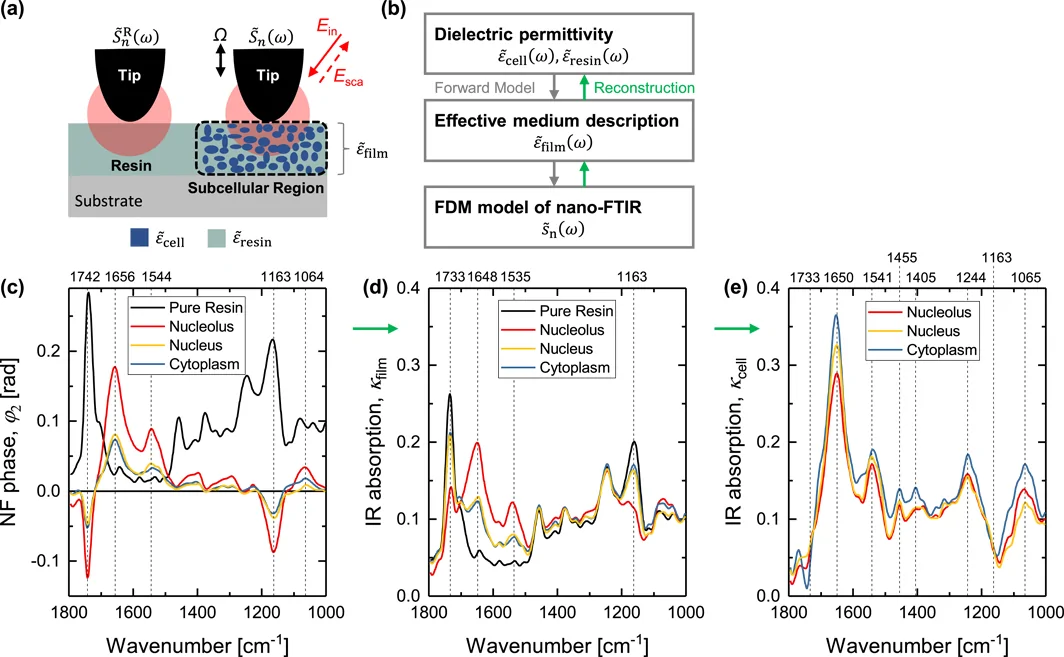
(a) Sketch of nano‐FTIR spectroscopy of resin‐embedded cell sections (b) model that relates the cell dielectric permittivity, ${\tilde{\varepsilon }}_{\text{cell}}\left(\omega \right)$, with the near‐field spectra, ${\tilde{s}}_{n}\left(\omega \right)$. (c) Referenced near‐field phase spectra of the three considered subcellular regions, $\varphi_{2}\left(\omega \right)$, from Figure 2e (colored lines). For comparison, the near‐field spectrum, $\varphi_{2}\left(\omega \right)$, of the pure resin is shown (black line). (d) Absorption index spectra of the thin film, $\kappa_{\text{film}}\left(\omega \right)=\text{Im}\left\{\sqrt{{\tilde{\varepsilon }}_{\text{film}}\left(\omega \right)}\right\}$. (e) Cell absorption index spectra, $\kappa_{\text{cell}}\left(\omega \right)=\text{Im}\left\{\sqrt{{\tilde{\varepsilon }}_{\text{cell}}\left(\omega \right)}\right\}$. See text for symbols. Peak positions are calculated as the median value across all spectra, see Supporting Information S1: Table S3 for band assignment.

We assume that the cell material (blue color) is finely dispersed within the resin embedding medium (teal color), which can be attributed to resin infiltration into the cell during sample preparation. Importantly, the fine structure of individual subcellular regions is assumed to be much smaller than the near‐field focus spot size (approx. 50 nm, set by the tip apex radius) such that it is not spatially resolved in the near‐field measurement. Thus, from the point of view of nano‐FTIR spectroscopy, the probed subcellular region can be described as a macroscopically homogeneous thin film of an effective dielectric permittivity, ${\tilde{\varepsilon }}_{\text{film}}\left(\omega \right)$. Specifically, we apply the Landau–Lifshitz–Looyenga effective medium theory to quantify ${\tilde{\varepsilon }}_{\text{film}}\left(\omega \right)$, which is valid for a finely dispersed mixture of two materials of similar refractive index,

(6) $${\tilde{\varepsilon }}_{\text{film}}\left(\omega \right)={\left[c\cdot {\tilde{\varepsilon }}_{\text{cell}}{\left(\omega \right)}^{\frac{1}{3}}+\left(1-c\right)\cdot {\tilde{\varepsilon }}_{\text{resin}}{\left(\omega \right)}^{\frac{1}{3}}\right]}^{3},$$

where ${\tilde{\varepsilon }}_{\text{cell}}\left(\omega \right)$ and ${\tilde{\varepsilon }}_{\text{resin}}\left(\omega \right)$ are the dielectric permittivity of the cell and resin material, respectively, and c the relative concentration of the cell material. Next, we apply a perturbative finite dipole model (FDM) for nano‐FTIR spectroscopy of thin films to quantitatively relate ${\tilde{\varepsilon }}_{\text{film}}\left(\omega \right)$ with the measured near‐field spectra, ${\tilde{s}}_{n}\left(\omega \right)$ [51]. This model takes into account the film thickness, the substrate material and instrumental settings, such as demodulation order, tapping amplitude and tip apex radius, and signal referencing, and is thus capable of accurately describing the slight blue shift of IR molecular vibration bands in nano‐FTIR spectra when compared to standard transmission FTIR spectra [42, 53].

Having defined the forward model that maps from the near‐field signal, ${\tilde{s}}_{n}\left(\omega \right)$, to cellular dielectric permittivity, ${\tilde{\varepsilon }}_{\text{cell}}\left(\omega \right)$ (gray arrows, Figure 4b), we now implement an inversion procedure to extract ${\tilde{\varepsilon }}_{\text{cell}}\left(\omega \right)$ from ${\tilde{s}}_{n}\left(\omega \right)$ (green arrows). First, the perturbative FDM is inverted to extract ${\tilde{\varepsilon }}_{\text{film}}\left(\omega \right)$ from ${\tilde{s}}_{n}\left(\omega \right)$. This inversion procedure is well documented and tested for nano‐FTIR spectroscopy of thin organic films [51]. Secondly, spectral decomposition of ${\tilde{\varepsilon }}_{\text{film}}\left(\omega \right)$ is implemented by iteratively solving Equation (6) to extract the cell dielectric permittivity, ${\tilde{\varepsilon }}_{\text{cell}}\left(\omega \right)$, and the relative concentration of the cell material in the probed sample volume, c (details see Methods). Finally, the cell dielectric permittivity, ${\tilde{\varepsilon }}_{\text{cell}}\left(\omega \right)$, is casted into the form of the absorption index spectrum, κ(ω), which is a simple description of the IR absorbance measured in standard transmission FTIR spectroscopy (valid for pure materials and ignoring any reflection losses) [52],

(7) $$A\left(\omega \right)\propto \omega \kappa_{\text{cell}}\left(\omega \right),$$

where $\kappa_{\text{cell}}\left(\omega \right)=\text{Im}\left\{\sqrt{{\tilde{\varepsilon }}_{\text{cell}}\left(\omega \right)}\right\}$. Note that chemical analysis based on κ(ω) is already established in (far‐field) IR ellipsometry studies of thin organic films [54, 55, 56]. $\kappa_{\text{cell}}\left(\omega \right)$ reflects the cell composition—a heterogeneous medium comprising proteins, sugar, DNA and other constituents [57]. $\kappa_{\text{cell}}\left(\omega \right)$ is independent of the material concentration, c, in Equation (6); therefore, we perform chemical interpretation of $\kappa_{\text{cell}}\left(\omega \right)$ without further normalization.

Figure 4c–e demonstrates the presented extraction process using the cell section data from Figure 2e. Importantly, the resin‐derived IR absorption lines are removed in $\kappa_{\text{cell}}\left(\omega \right)$ (Figure 4e), thus solving this major challenge with resin‐embedded cell sections and allowing for quantification of IR data. This removal can be recognized most prominently by the absence of the resin peaks near 1742 and $1163\;{\text{cm}}^{-1}$. As a result, the expected IR absorption bands of the cell are now revealed with clarity in the 1500–$1100\;{\text{cm}}^{-1}$ spectral region, contributing to a more complete biochemical picture. Specifically, peaks are distinctly observed at $1455\;{\text{cm}}^{-1}$ for all three subcellular regions (methylene deformations), whereas a significant $1405\;{\text{cm}}^{-1}$ band is only observed for the cytoplasm (symmetric stretching vibrations of the $\text{CO}O^{-}$ group of fatty and amino acids and OH deformations of ribose ring in DNA/RNA). The $1244\;{\text{cm}}^{-1}$ band is now clearly revealed ($\nu_{\text{as}}PO_{2}^{-}$, asymmetric phosphate stretching vibration), which agrees well with the expected band position for RNA and A‐DNA [50]. Those bands were previously camouflaged by the IR absorption peaks of the resin in this spectral region (c.f. Figure 4c,e).

Further, reconstruction of $\kappa_{\text{cell}}\left(\omega \right)$ recovers peak positions that are consistent with standard transmission FTIR spectroscopy. For the Amide I and II bands, where resin IR absorption is minimal, peaks shift to lower wavenumbers from 1656 and $1543\;{\text{cm}}^{-1}$ in $\varphi_{2}\left(\omega \right)$ (Figure 4c) to 1650 and $1541\;{\text{cm}}^{-1}$ in $\kappa_{\text{cell}}\left(\omega \right)$ (Figure 4f), respectively, as expected [42]. This downward shift is consistent across all four cells (Supporting Information S1: Table S4). For the $1064\;{\text{cm}}^{-1}$ band, where resin IR absorption is significant, the peak shifts slightly in the opposite direction from $1064\;{\text{cm}}^{-1}$ in $\varphi_{2}\left(\omega \right)$ to $1065\;{\text{cm}}^{-1}$ in $\kappa_{\text{cell}}\left(\omega \right)$. This upward shift is a result of the disentanglement of resin and cell absorption that is provided by our effective medium extension to the FDM model.

Figure 5a–d summarizes the reconstructed spectra, $\kappa_{\text{cell}}\left(\omega \right)$, for all four considered cells. Table 1 summarizes the corresponding cell material concentration, c. We quantify the peak intensity of all identified molecular vibration bands as they appear in $\kappa_{\text{cell}}\left(\omega \right)$ in Figure 5e, and peak positions are assigned in Supporting Information S1: Table S3. Note that we observe small variations in the Amide I and II peak positions in $\kappa_{\text{cell}}\left(\omega \right)$ across the four cells ($\leq \;6\;{\text{cm}}^{-1}$), Figure 5a–d. The presented model is not the cause for these variations. They are already present in the measured near‐field spectra, $\varphi_{2}\left(\omega \right)$ (Figure 3) and are attributed to instrument noise as well as genuine differences between cell lines. The model simply propagates these variations to $\kappa_{\text{cell}}\left(\omega \right)$. The reconstructed spectra $\kappa_{\text{cell}}\left(\omega \right)$ also show a small peak near $1730\;{\text{cm}}^{-1}$, but this is not a cell‐related feature, Figure 5c–d. This peak is assigned to the C=O vibration of the resin material at $1730\;{\text{cm}}^{-1}$ in $\kappa_{\text{film}}\left(\omega \right)$ (black line in Figure 4d), which is not fully suppressed in the cell absorption index spectra, $\kappa_{\text{cell}}\left(\omega \right)$.

**FIGURE 5:**
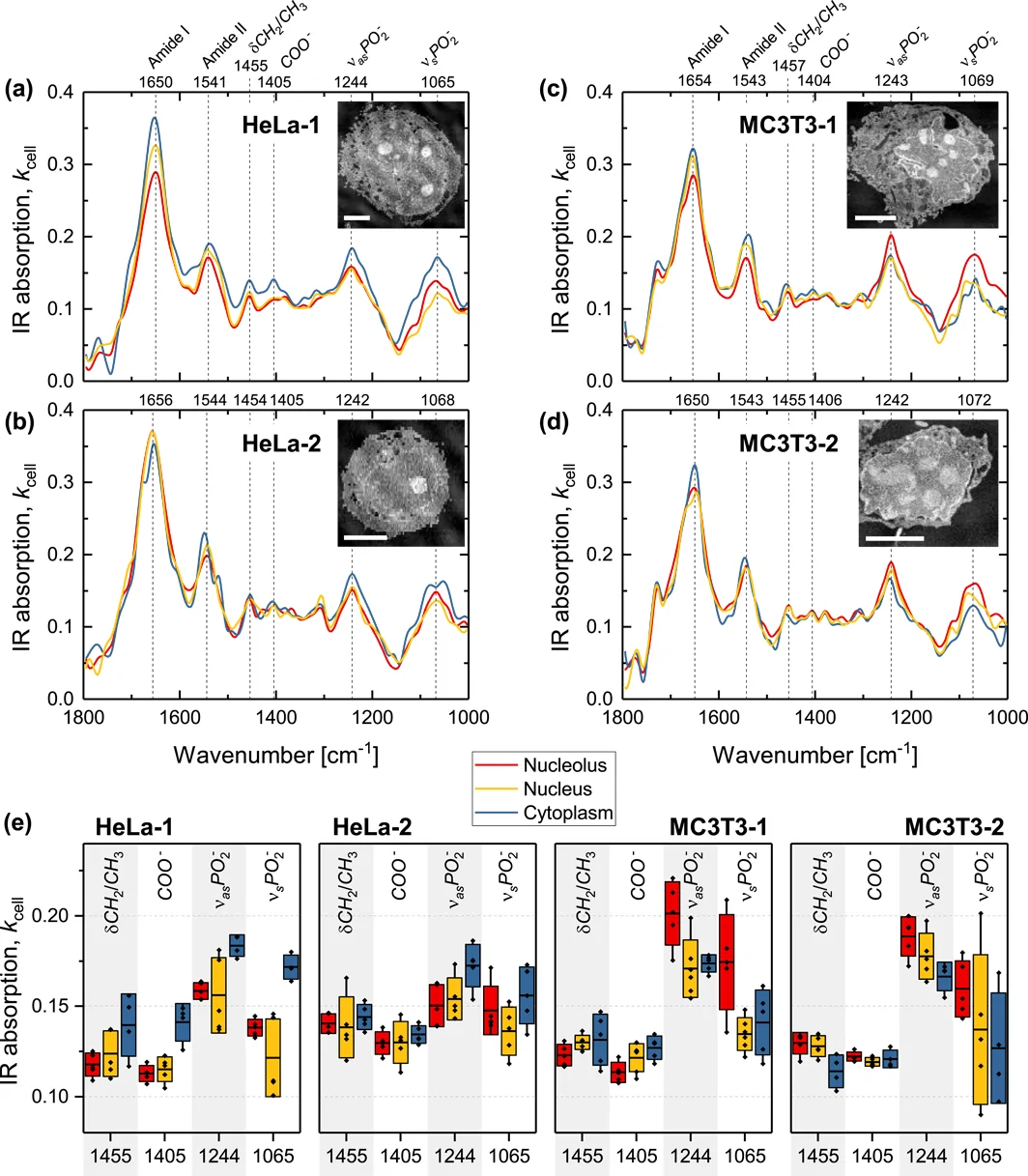
(a–d) IR absorption index spectra, $\kappa_{\text{cell}}\left(\omega \right)$, of the two HeLa and two MC3T3 cells. Insets show the near‐field phase images, $\varphi_{3}$, of the cells taken at the Amide I band ($1660\;{\text{cm}}^{-1}$). The scale bar is 5μm. (e) Visualization of peak intensity with wavenumber as categorical labels to quantify the chemical differences between the subcellular regions of nucleolus, nucleus, and cytoplasm for the four cells from (a–d). Whisker plots correspond to the standard deviation and the midline represents the sample mean value, datapoints are superposed in black.

**TABLE 1:** The retrieved composition of cell material, c, at the three considered subcellular regions.

Cell material fraction, c,	… in cytoplasm [%]	… in nucleus [%]	… in nucleolus [%]	Ratio nucleolus/nucleus
HeLa‐1	22±3	26±3	61±7	2.31±0.35
HeLa‐2	28±4	28±3	54±4	1.90±0.24
MC3T3‐1	47±3	45±9	63±14	1.38±0.40
MC3T3‐2	43±8	46±6	67±7	1.45±0.24

*Note:* The remainder is made of resin with a relative concentration of 1−c. For example, the cytoplasm subcellular region of HeLa‐1 contains 22% cell material and 78% resin material. Generally, the nucleolus is about twice as dense as the cytoplasm and nucleus. The resin concentration was found to be as high as 78% within some cellular parts contained inside of the cell sections, indicating that the cell material is significantly diluted in the less denser regions of the cell section, especially in the cytoplasm and the nucleus, whereas the nucleoli are found to be denser by a factor of 1.90–2.31 in case of HeLa and 1.38–1.45 in case of MC3T3 cells.

We note in passing that the weak resin absorption between 1680 and $1480\;{\text{cm}}^{-1}$ provides a spectral window for unobstructed measurement of cell absorption (black line in Figure 4c). Particularly, the Amide I and II bands can be measured accurately and are thus well suited for single‐wavelength s‐SNOM imaging of cell sections. Outside this spectral range, the influence of the resin absorption becomes more prominent, reducing near‐field phase contrast and causing contrast inversion in extreme cases (Supporting Information S1: Figure S11).

## 3 Discussions

Nano‐FTIR spectroscopy of cell sections enables biological interpretation in analogy to standard transmission IR spectroscopy. The most salient finding from our data is the pronounced variation in material concentration within the cell. Variation in protein content is revealed qualitatively by the contrast in the Amide I images (Figure 2b) as well as by the peak height in the unnormalized nano‐FTIR spectra (Figure 2e and Supporting Information S1: Figure S10). Our model further quantifies the overall material concentration at the considered subcellular locations (Table 1). Notably, the nucleolus was found to be denser than the rest of the nucleus by a factor of about 1.9–2.3 in case of the HeLa cells and 1.38–1.45 in case of the MC3T3 cells (Table 1). These values are consistent with prior diffraction‐limited optical microscopy studies that suggested that the nucleolus is approximately twice as mass dense as the surrounding nucleoplasm [58, 59]. Therefore, nano‐FTIR spectroscopy offers a new approach for assessing material concentration within cells that is based on the quantitative measurement of the sample optical properties rather than measurement of the intensity of labels or stains in the sample as it is the case with established methods.

By reconstructing the cellular absorption index spectra, $\kappa_{\text{cell}}\left(\omega \right)$, the resin contribution to the nano‐FTIR spectra is removed and chemical differences across the cells can be evaluated. Small yet consistent spectral differences across the four cells (Figure 5). This result aligns with expectations from standard transmission FTIR spectroscopy [8], but this is now directly confirmed at nanoscale spatial resolution using nano‐FTIR spectroscopy. The most notable differences appear in the asymmetric and symmetric P–O stretching vibrations at near 1244 and $1065\;{\text{cm}}^{-1}$, respectively, which allow for tracing nucleic acid spectral features across the cell section. Specifically, nucleoli showed higher intensity in the P–O bands compared to the nucleus for both cell lines (Figure 5e), reflecting the localized enrichment of nucleic acids. Although the nucleus itself shows comparable P–O band intensities in HeLa and MC3T3 cells, the nucleolus, however, exhibits significantly stronger P–O bands in MC3T3 cells. This observation differs from prior studies, which suggest that HeLa cell nuclei exhibit higher nucleic acid content [60]. On the other hand, the discrepancies may also be explained by the HeLa's larger nuclear volume and differences in detection methods (fluorescence‐based methods vs. the presented nano‐FTIR approach), thus warranting further exploration. Additionally, nucleic acid bands in the cytoplasm were found to be dependent on the cell type: HeLa showed elevated P–O bands in the cytoplasm (blue color in Figure 5e), whereas MC3T3 showed similar to slightly reduced P–O bands of cytoplasm (blue) when compared to nucleus (yellow). Note that identifying DNA conformation from peak positions is still limited by the instrument spectral resolution of $12.5\;{\text{cm}}^{-1}$ and noise levels, but future enhancements to nano‐FTIR spectroscopy could make this feasible and should be pursued.

From an application perspective, nano‐FTIR spectroscopy provides multichannel data at individual cellular sites, providing a basis for detailed biochemical interpretation. Techniques previously applied to isolated samples may now be extended to resin‐embedded cell sections, including (i) structural analysis of proteins as biomarkers for disease [14, 61] and (ii) monitoring drug delivery [62]. In contrast to standard transmission FTIR spectroscopy, nano‐FTIR spectroscopy offers a visual approach to molecular mapping that does not require extraction of cell material (such as in cell fractionation studies) and conserves the spatial cellular distribution. By combining this direct measurement on the subcellular scale with the analytical power of IR spectroscopy—such as discrimination of RNA and DNA [63]—, nano‐FTIR spectroscopy could significantly expand the scope of ultrastructural cell investigations. For context, Raman spectroscopy [64] and optical‐photothermal infrared (O‐PTIR) spectroscopy [65] provide 250–400 nm spatial and ∼1 μm vertical resolution, making them useful tools for probing larger subcellular regions and for noncontact screening‐type applications. For applications requiring nanoscale resolution—such as recognition of small subcellular structures, tracking drug delivery, and nanoparticle uptake—the combination of nano‐FTIR spectroscopy (20–50 nm spatial resolution) with thin cell sections (∼80 nm vertical resolution) is especially advantageous.

From a technical perspective, resin‐embedded cell cuts provide improved signal consistency in nano‐FTIR spectroscopy when compared to studies of whole (dried) cells. Their smooth surface enables reliable AFM operation, and their uniform thickness ensures consistent tip‐sample coupling [53] and thus provides for more stable and interpretable s‐SNOM signals. Moreover, signal referencing to the cell‐adjacent resin material avoids negative phase contrast [66], eliminates signatures of the PDMS‐contamination of the AFM tip [67] and suppresses indirect illumination effects [68] due to the matched refractive indices of the resin and cellular materials. The main disadvantage of using resin‐embedded cell cuts are the strong IR absorption lines of the resin material. To address this challenge, we presented a model to remove the resin lines and this approach should be validated further in the future. Thus, we anticipate that resin‐embedded sample cuts can be a viable route for the chemical mapping of the nanostructure of many soft matter samples in general.

Further, our data highlight that resin‐embedded cell sections are challenging samples for current IR nano‐imaging approaches because of the following compounding factors: (i) the relatively weak IR absorption of biological cell material, especially in the lower part of the fingerprint region spanning 1000–1450 ${\text{cm}}^{-1}$, (ii) the need to sensitively measure small changes in the IR absorption for the intended ultrastructural analysis based on chemical contrast, and (iii) the aggravating fact that the cell material is diluted by up to 80% by the embedding resin material, thus further reducing the IR absorption signal. We overcame this challenge by developing and applying interleaved referencing for nano‐FTIR spectroscopy, which reduced noise in the near‐field phase spectra down to 4 mrad (∼0.2°) and allowed to resolve weak cell IR absorption peaks with clarity. Thus, high‐quality spectra at the limit of the instrument noise level could be obtained from very small cell volumes of an estimated size of 50 nm × 50 nm × 80 nm ($2\cdot 10^{-4}\;{\mu m}^{3}$), amounting to only a small (1/25,000) part of the entire cell volume (nominal $5\;{\mu m}^{3}$). This high sensitivity can be attributed to the combined effect of field enhancement at the apex of the AFM tip, the use of high‐brilliance broadband laser sources and the demodulation techniques used in nano‐FTIR spectroscopy [41].

## 4 Conclusions

We have presented a method for high‐sensitivity nano‐FTIR spectroscopy for the chemical analysis of cellular ultrastructure that uniquely combines resin‐embedded ultramicrotomed cell samples, interleaved referencing, and data reconstruction based on the effective medium description. With this approach, we showed that it is possible to reveal the small but consistent IR spectral differences between subcellular regions in human (HeLa) and mouse (MC3T3) cell lines, allowing to observe differences in the nucleic acid (DNA/RNA) content across the cell. Our method can assess chemical information in a direct and label‐free manner at a spatial resolution of 50 nm and using the contrast provided by the intrinsic molecular vibration. This offers an advantage over established methods that either rely on indirect measurements (e.g., cell fractionation IR studies) or applying labels (as in fluorescence or electron microscopy). The spectral information obtained with nano‐FTIR is amenable to biological interpretation, which could enable to monitor the occurrence of disease, drug delivery, and nanoparticle treatments at the nanoscale within the cell. We believe that this study can establish nano‐FTIR spectroscopy as a valuable tool for the label‐free ultrastructural microscopy for soft matter science, particularly at the interface of nanoscience and life science.

## 5 Methods

### 5.1 Sample Preparation

The cells were prepared following a standard protocol for scanning electron microscopy (SEM) using Sorenson’s buffer at pH 7.4. After chemical fixation in 4% formaldehyde for 30 min, the cells were mildly centrifuged at 800g for 2 min, washed with Sorenson’s and the final pellet was embedded in 2% agarose and left to cool. Afterward, agarose cuts were immersed in 1% Osmium tetraoxide for 30 min and subsequently washed. The following dehydration series was performed in steps of 10 min each with EtOH/Sorenson's v/v: 30% → 50% → 70% → 90% → 100%, followed by acetone substitution for 20 min. Infiltration in Spurr’s resin was carried out in steps of 20 min each where the samples were inserted in acetone/Spurr's v/v: 1/1; 1/2; and 0/1. Polymerization took place in BEEM embedding capsules placed in a vacuum oven at 60°C for 72 h. Slices of nominal 80 nm thickness are cut using ultramicrotome Leica EM UC7 (Leica Microsystems, L'Hospitalet de Llobregat, Spain) equipped with a diamond knife (DiATOME, Quakertown, PA, USA), and the flakes were deposited on a clean Si substrate.

### 5.2 Nano‐FTIR Spectroscopy

We provide further details to the nano‐FTIR spectroscopy setup (neaSNOM and Attocube Systems GmbH) described in Figure 1. The interferogram length was 400μm, corresponding to a spectral resolution of $12.5\;{\text{cm}}^{-1}$. Four interferograms were recorded at 1024 points at an integration time of 32 ms and subsequently averaged per measurement. Interleaved referencing was performed by taking eight measurements per location on the cell (C) and nine measurements on the nearby resin (R). Total acquisition time per ROI on the sample was 39 min to ensure that a sensitivity of 4 mrad was reached in the near‐field phase spectrum, $\varphi_{2}\left(\omega \right)$. The individual spectra were then processed as follows: (i) apodization of the nano‐FTIR interferograms using a flattop window, (ii) performing the Fourier transform, and (iii) referencing the individual spectra as described in the main text. Signal averaging was then performed on the individual referenced spectra as in Equation (3). To cover the fingerprint spectral range, nano‐FTIR spectroscopy was performed for two different laser configurations using configuration B (offering a bandwidth of 900 to $1500\;{\text{cm}}^{-1}$) and C (1200 to $1800\;{\text{cm}}^{-1}$) in the microscope software. Stitching of the individual near‐field spectra was applied as documented in ref. [14]. The final spectra were interpolated using the spline method to determine the peak position and shape of the sample IR absorption bands with accuracy. The exact locations (ROIs) for performing nano‐FTIR spectroscopy within the cell section are specified in the Supporting Information S1: Note S3.

### 5.3 Extraction of Cell IR Absorption

We applied a perturbative finite dipole model (FDM) for nano‐FTIR spectroscopy of thin films on a substrate, which takes into account aspects of tip vibration, signal demodulation, and signal referencing [51]. This model is thus capable of providing quantitative accuracy in describing the spectral signature of molecular vibrational in the near‐field spectra. The perturbative FDM particular model is reasonably accurate for film thicknesses in the range between 10 and 150 nm, requires fewer empirical parameters compared to other thin‐layer models and further allows for direct inversion of the thin film dielectric permittivity from near‐field spectra [51, 69]. In detail, the light scattering from the tip is described by the scattering coefficient, $\tilde{\sigma }={\tilde{E}}_{\text{sca}}/E_{\text{in}}$, which relates the tip‐scattered field, ${\tilde{E}}_{\text{sca}}$, to the incident field $E_{\text{in}}$. The incident field, $E_{\text{in}}$, polarizes the tip, which yields an effective dipole moment $\tilde{p}={\tilde{\alpha }}_{\text{eff}}\left(1+\tilde{r}\right)E_{\text{in}}$, where ${\tilde{\alpha }}_{\text{eff}}$ is the effective polarizability of the tip that accounts for the near‐field coupling between the tip and the sample (the tilde indicates complex‐valued quantities) and $\tilde{r}$ is the far‐field reflection coefficient of the sample surface. The field backscattered by the tip can be described as ${\tilde{E}}_{\text{sca}}=\left(1+\tilde{r}\right)\tilde{p}$. The scattering coefficient can then be written as follows:

(8) $$\tilde{\sigma }={\tilde{\alpha }}_{\text{eff}}{\left(1+\tilde{r}\right)}^{2}.$$

A solution for the effective tip polarizability, ${\tilde{\alpha }}_{\text{eff}}$, can be obtained in the electrostatic approximation, which relates ${\tilde{\alpha }}_{\text{eff}}$ with the sample dielectric permittivity, $\tilde{\varepsilon }$:

(9) $${\tilde{\alpha }}_{\text{eff}}=C\left(1+\frac{1}{2}\cdot \frac{f_{0}\left(H\right)\beta \left(\tilde{\varepsilon }\right)}{1-f\left(H\right)\beta \left(\tilde{\varepsilon }\right)}\right).$$

where $f_{0}\left(H\right)$ and f(H) are model‐specific functions describing the effect of the tip‐sample distance, H, and $\beta \left(\tilde{\varepsilon }\right)=\left(\tilde{\varepsilon }-1\right)/\left(\tilde{\varepsilon }+1\right)$ is the quasistatic reflection coefficient of the semi‐infinite half‐space made of the sample material and C is a distance‐independent constant.

To model a thin film on a substrate, the perturbative FDM describes the tip‐sample interaction in form of multiple scattering events between the tip, the thin film, and the substrate. Each individual scattering event considers that the tip is interacting via the near field with a virtual half‐space occupied by either the thin film material ($\tilde{\varepsilon }$) or the substrate material ($\varepsilon_{\text{sub}}$), beginning at z=0 or z=−d. Specifically, the following three first order interaction terms are obtained: (i) the scattering tensor, ${\hat{T}}_{\text{sca}}\left(\varepsilon_{\text{sub}},d\right)$, describing the scattering off the substrate at z=−d, (ii) the scattering tensor, ${\hat{T}}_{\text{sca}}\left(\tilde{\varepsilon },0\right)$, describing the scattering off the virtual half‐space occupied by thin film material at z=0, and (iii) the scattering tensor, ${\hat{T}}_{\text{sca}}\left(\tilde{\varepsilon },d\right)$, describing the scattering off the virtual half‐space occupied by thin film material at z=−d. Summation over the individual scattering events in form of a Born series expansion yields the total near‐field interaction of the tip with a thin film on a substrate (see also Figure 3 in ref. [51]),

(10) $${\hat{T}}_{\text{sca}}={\hat{T}}_{\text{sca}}\left(\varepsilon_{\text{sub}},d\right)+{\hat{T}}_{\text{sca}}\left(\tilde{\varepsilon },0\right)-{\hat{T}}_{\text{sca}}\left(\tilde{\varepsilon },d\right)+\text{SO}+\text{HO}.$$

The perturbative FDM for thin films considered terms up to second interaction order, SO, and truncated higher order terms, HO, for practical purposes. The resulting effective polarizability is then expressed as a sum of a total of 11 terms

(11) $$\alpha_{\text{eff}}=C\left[2+\xi_{0}\left(\varepsilon_{\text{sub}},d\right)+\xi_{0}\left(\tilde{\varepsilon },0\right)-\xi_{0}\left(\tilde{\varepsilon },d\right)+2\xi_{1}\left(\tilde{\varepsilon },d\right)\xi_{0}\left(\tilde{\varepsilon },d\right)-\left(\xi_{1}\left(\tilde{\varepsilon },0\right)\xi_{0}\left(\tilde{\varepsilon },d\right)+\xi_{1}\left(\tilde{\varepsilon },d\right)\xi_{0}\left(\tilde{\varepsilon },0\right)\right)+\left(\xi_{1}\left(\tilde{\varepsilon },0\right)\xi_{0}\left(\varepsilon_{\text{sub}},d\right)+\xi_{1}\left(\varepsilon_{\text{sub}},d\right)\xi_{0}\left(\tilde{\varepsilon },0\right)\right)-\left(\xi_{1}\left(\tilde{\varepsilon },d\right)\xi_{0}\left(\varepsilon_{\text{sub}},d\right)+\xi_{1}\left(\varepsilon_{\text{sub}},d\right)\xi_{0}\left(\tilde{\varepsilon },d\right)\right)\right],$$

with

(12) $$\xi_{0}\left(\tilde{\varepsilon },d\right)=\frac{f_{0}\left(H+d\right)\beta \left(\tilde{\varepsilon }\right)}{1-f\left(H+d\right)\beta \left(\tilde{\varepsilon }\right)},\xi_{1}\left(\tilde{\varepsilon },d\right)=\frac{f\left(H+d\right)\beta \left(\tilde{\varepsilon }\right)}{1-f\left(H+d\right)\beta \left(\tilde{\varepsilon }\right)},$$

and

(13) $$f_{0}\left(H\right)=\left(\tilde{g}-\frac{2H+W_{0}+R_{t}}{2L}\right)\frac{\ln\frac{4L}{4H+2W_{0}+R_{t}}}{\ln\frac{4L}{R_{t}}},$$

(14) $$f\left(H\right)=\left(\tilde{g}-\frac{2H+W_{i}+R_{t}}{2L}\right)\frac{\ln\frac{4L}{4H+2W_{1}+R_{t}}}{\ln\frac{4L}{R_{t}}},$$

where $W_{0}\approx 1.31\;R_{t}$ and $W_{i}\approx R_{t}/2$ with $R_{t}$ being the tip apex radius. Vertical tip vibration, H=H(t), at frequency Ω and subsequent signal demodulation at a higher harmonic, nΩ, are considered by calculation of the n‐th Fourier coefficient of the scattering coefficient,

(15) $${\tilde{s}}_{n}\left(\omega \right)\propto \sigma_{n}\left(\beta \right)={\hat{F}}_{n}\left[\sigma \left(\beta ,H\left(t\right)\right)\right]=\int_{0}^{T}dt\;\sigma \left(\beta ,H\left(t\right)\right)\;e^{\text{in}\Omega t}.$$

The n‐th order demodulated detector signal in the experiment, ${\tilde{s}}_{n}\left(\omega \right)$, is proportional to $\sigma_{n}\left(\beta \right)$. The above approximation (Equation 11) yields accurate results for thin films made of weak molecule oscillators, such as polymers, biological material, and other materials, with sufficiently small quasistatic reflection coefficient, β<1. In case of the cell sections, the far‐field reflection factor is neglected, $\tilde{r}=0$, because the refractive index of resin and cell materials is very similar, thus the expected far‐field reflection coefficient is also similar on both materials and cancels out in performing the signal referencing. The constant $C\propto W_{0}^{2}E_{\text{in}}$ cancels out after normalizing the signal to that of a reference material.

To perform the inversion of ${\tilde{s}}_{n}\left(\omega \right)$ and extract ${\tilde{\varepsilon }}_{\text{film}}\left(\omega \right)$, the tip polarizability, ${\tilde{\alpha }}_{\text{eff}}$ (Equation 11), is expanded into a Taylor series in powers of ${\tilde{\beta }}_{\text{film}}$ to the 6‐th order,

(16) $${\tilde{\alpha }}_{\text{eff}}=\sum_{j=0}^{6}{\tilde{\alpha }}_{j}{\left({\tilde{\beta }}_{\text{film}}\right)}^{j},$$

where ${\tilde{\alpha }}_{j}$ are the expansion coefficients. The referenced near‐field spectrum, ${\tilde{s}}_{n}\left(\omega \right)$, can then be described by

(17) $${\tilde{s}}_{n}\left(\omega \right)=\sum_{j=0}^{6}{\left({\tilde{\beta }}_{\text{film}}\left(\omega \right)\right)}^{j}\frac{{\hat{F}}_{n}\left[{\tilde{\alpha }}_{j}\right]}{{\hat{F}}_{n}[{\tilde{\alpha }}_{\text{eff}}^{R}\left(\omega \right)]},$$

where tip vibration and signal demodulation is considered by taking the n‐th Fourier coefficient, ${\hat{F}}_{n}\left[{\tilde{\alpha }}_{j}\right]$, of the expansion coefficients, ${\tilde{\alpha }}_{j}$, with respect to the tip vibration. Signal referencing is implemented by dividing ${\hat{F}}_{n}\left[{\tilde{\alpha }}_{j}\right]$ by the n‐th Fourier coefficient, ${\hat{F}}_{n}\left[{\tilde{\alpha }}_{\text{eff}}^{R}\right]$, where the tip‐polarizability spectrum, ${\tilde{\alpha }}_{\text{eff}}^{R}\left(\omega \right)$, is calculated using the perturbative FDM for the resin film on top of the Si substrate. To this end, the dielectric permittivity of the resin film, ${\tilde{\varepsilon }}_{\text{resin}}\left(\omega \right)$, was measured in a calibration experiment. Equation (17) represents a simple polynomial equation for ${\tilde{\beta }}_{\text{film}}\left(\omega \right)$ and is easily invertible. To this end, the roots of the polynomial coefficients, ${\hat{F}}_{n}\left[{\tilde{\alpha }}_{j}\right]$, are calculated and the following solutions for ${\tilde{\beta }}_{\text{film}}$ are discarded for each frequency point in ω: $\text{Re}\left\{{\tilde{\beta }}_{\text{film}}\right\}<0$, $\text{Re}\left\{{\tilde{\beta }}_{\text{film}}\right\}>1$, $\text{Im}\left\{{\tilde{\beta }}_{\text{film}}\right\}<0$, and $\text{Im}\left\{{\tilde{\beta }}_{\text{film}}\right\}>\text{Re}\left\{{\tilde{\beta }}_{\text{film}}\right\}$. The remaining solution for ${\tilde{\beta }}_{\text{film}}$ is then saved, and the film dielectric permittivity is calculated, ${\tilde{\varepsilon }}_{\text{film}}\left(\omega \right)=\left({\tilde{\beta }}_{\text{film}}\left(\omega \right)+1\right)/\left(1-{\tilde{\beta }}_{\text{film}}\left(\omega \right)\right)$. The following parameters were used: tip apex radius R=50nm, tip half length L=600nm, tapping amplitude A=107nm, film thickness d=80nm, substrate permittivity $\varepsilon_{\text{subs}}=12$ (describing Si at mid‐IR frequencies), model parameter $g=0.7e^{0.06i}$, and demodulation order n=2.

Next, the Landau–Lifshitz–Looyenga effective medium description is applied to extract the cell dielectric permittivity, ${\tilde{\varepsilon }}_{\text{cell}}\left(\omega \right)$, from the film dielectric permittivity, ${\tilde{\varepsilon }}_{\text{film}}\left(\omega \right)$. To this end, we solve Equation (6) iteratively. As an initial guess for ${\tilde{\varepsilon }}_{\text{cell}}\left(\omega \right)$, the dielectric permittivity of Matrigel is assumed, a membrane protein that is a first approximation for a biological cell in conventional FTIR spectroscopy, which is available in tabulated form from literature [70]. Next, Equation (6) is evaluated for ${\tilde{\varepsilon }}_{\text{cell}}\left(\omega \right)$ (initial guess), ${\tilde{\varepsilon }}_{\text{resin}}\left(\omega \right)$ (obtained in a calibration experiment on the same sample) and a range of values for the cell concentration, c=0..1 to obtain a prediction for the film dielectric permittivity, ${\tilde{\varepsilon }}_{\text{film}}^{\text{pred}}\left(\omega \right)$. The concentration c is saved for which the deviation from the ${\tilde{\varepsilon }}_{\text{film}}\left(\omega \right)$ is smallest, $\Delta {\tilde{\varepsilon }}_{\text{film}}\left(\omega \right)={\tilde{\varepsilon }}_{\text{film}}^{\text{pred}}\left(\omega \right)-{\tilde{\varepsilon }}_{\text{film}}\left(\omega \right).$ Subsequently, ${\tilde{\varepsilon }}_{\text{cell}}\left(\omega \right)$ is updated by ${\tilde{\varepsilon }}_{\text{cell}}\left(\omega \right):={\tilde{\varepsilon }}_{\text{cell}}\left(\omega \right)-\Delta {\tilde{\varepsilon }}_{\text{film}}\left(\omega \right)\cdot 0.05/c$. This process is repeated until convergence is achieved, $\int_{\omega }\left|\Delta {\tilde{\varepsilon }}_{\text{film}}\left(\omega \right)\right|<10^{-3}$. A MATLAB script is made available with this study that details the implementation of this reconstruction method.

### 5.4 Electron Microscopy

The cell sections were measured with scanning electron microscopy (SEM) on the FEI Helios Nanolab/FEI Helios 450S. The imaging settings were electron beam voltage 2 kV and current 0.40 nA, working distance 4.3 mm and using the CBS (Concentric Backscatter) detector. Electron microscopy is destructive to molecular bonds in the sample so that SEM was performed after s‐SNOM imaging and nano‐FTIR spectroscopy work had been completed.

## Author Contributions

**Florina Marxer:** conceptualization, methodology, data curation, investigation, writing – original draft, writing – review and editing. **Marta Gallego:** methodology, writing – review and editing. **Carlos Sanchez‐Cano** **:** methodology, writing – review and editing, supervision, funding acquisition. **Tomasz P. Wrobel** **:** data curation, writing – review and editing. **Wolfgang J. Parak:** writing – review and editing, supervision, funding acquisition. **Rainer Hillenbrand:** methodology, investigation, writing – review and editing, supervision, funding acquisition. **Martin Schnell:** conceptualization, methodology, data curation, investigation, writing – original draft, writing – review and editing, supervision, funding acquisition.

## Conflicts of Interest

R.H. is a cofounder of Neaspec GmbH, which now is a part of Attocube Systems GmbH, a company producing s‐SNOM systems, such as the one used in this study. The remaining authors declare no conflicts of interest.

## Supporting information


Supporting Information S1

## Data Availability

The data underlying this study and the reconstruction script developed in this study are openly available in Zenodo repository at 10.5281/zenodo.15789575.
