# Supplementary material for: Infrared Nano‐Spectroscopy of Single Cell Sections at the Ultrastructural Level
Source: Nanophotonics. 2026 Mar 24;15(6):e70056. doi: 10.1002/nap2.70056 (PMC13642522; doi:10.1002/nap2.70056)
Supplement: Supplementary file 1 [file NAP2-15-e70056-s001.pdf]

Supplementary Information for
Infrared nano-spectroscopy of single cell sections at the ultrastructural level

Florina Marxer,[1] Marta Gallego,[2] Carlos Sanchez Cano,[3,4,5] Tomasz P. Wrobel,[6] Wolfgang J. Parak,[7] Rainer Hillenbrand,[1,4,8] and Martin Schnell[1,3,4*]

[1] CIC nanoGUNE BRTA, 20018 Donostia-San Sebastián, Spain

[2] Center for Cooperative Research in Biomaterials (CIC biomaGUNE), Basque Research and Technology Alliance (BRTA), Paseo de Miramon 182, San Sebastián, 20014, Spain.

[3] Donostia International Physics Center, Donostia-San Sebastian 20018, Spain

[4] IKERBASQUE, Basque Foundation for Science, 48013 Bilbao, Spain

[5] Polimero eta Material Aurreratuak: Fisika, Kimika eta Teknologia, Kimika Fakultatea, Euskal Herriko Unibertsitatea UPV/EHU, 20018 Donostia-San Sebastian, Spain

[6] Solaris National Synchrotron Radiation Centre, Jagiellonian University, ul. Czerwone Maki 98, 30-392 Krakow, Poland

[7] Universität Hamburg, 22761 Hamburg, Germany

[8] Department of Electricity and Electronics, UPV/EHU, 20018 Donostia-San Sebastián, Spain

**schnelloptics@gmail.com*

Supplementary Note 1: Interleaved referencing suppresses instrument drift

In Fig. S1, we show the effect of instrument drift in nano-FTIR spectroscopy of cell sections as it occurs during a typical measurement day and show how it can be eliminated by interleaved referencing. To this end, we use the data set of the MC3T3-2 cell presented in the main text, which consists of near-field spectra obtained at 16 locations on the cell.

We first show traditional referencing, where cell spectra, $\tilde{S}_{n,l}(\omega)$, and reference spectra, $\tilde{S}^{\mathrm{R}}_{n,l}(\omega)$, are recorded sequentially and averaging of the cell and reference spectra is done separately (Fig. S1(e)). Signal referencing is then done according to Eq. 1 of the main text, yielding a referenced near-field spectrum,

$$\tilde{s}_n(\omega) = s_n(\omega)e^{i\varphi_n(\omega)} = \tilde{S}_n(\omega)/\tilde{S}^{\mathrm{R}}_n(\omega). \quad (1)$$

Traditionally, a common reference spectrum, $\tilde{S}^{\mathrm{R}}_n(\omega)$, is used for all measurements, which we here simulated by calculating the average of all individual reference spectra contained in the MC3T3-2 data set. The so-obtained near-field phase spectra, $\varphi_2(\omega)$, of all 16 locations on the cell show a significant baseline (Fig. S1(a)). This is a typical observation in nano-FTIR spectroscopy and it is attributed to temperature fluctuations in the environment, which cause a slow drift in the optical path length and the alignment of the nano-FTIR interferometer. The time trace in Fig. S1(d), yellow line, quantifies that the average phase drift was about $2\ \mathrm{rad}$ during the course of the measurement day. Importantly, this phase drift exceeds the weak molecular absorption bands of cell sections and must thus be corrected ($\sim 0.2\ \mathrm{rad}$ for the Amide I band, see Fig. 2(e) in the main text). Typically, instrument drift is corrected by subtraction of a linear baseline from the near-field phase spectra. This procedure works well in case of isolated IR absorption bands (e.g. pure polymer samples), where frequency points with known zero IR absorption can serve as anchor points. However, this procedure is less effective in case of cell samples because those show significant IR absorption across the entire fingerprint spectral region. To illustrate this, we apply a linear fit to the cell spectra in the range between 1200 and 1700 cm^{-1} and subtract it from the cell spectra. As it can be recognized with Fig. S1(b), this correction procedure removes valid IR absorption information and yields reduced peak heights of molecular vibration bands (Fig. S1(b)).

In comparison, in interleaved referencing, cell spectra, $\tilde{S}_{n,l}(\omega)$, and reference spectra, $\tilde{S}^{\mathrm{R}}_{n,l}(\omega)$ are recorded in an interleaved fashion and signal averaging is performed over the referenced spectra, $\tilde{s}_{n,l}(\omega)$, as obtained by Eq. (4) in the main text

$$\tilde{s}_{n,l}(\omega) = \frac{\tilde{S}_{n,l}(\omega)}{\left[\tilde{S}^{\mathrm{R}}_{n,l}(\omega) + \tilde{S}^{\mathrm{R}}_{n,l+1}(\omega)\right]/2}, \quad (2)$$

and illustrated in Fig. S1(f). We note that for clarity we show a simplified schematic of interleaved referencing in Fig. S1(f) and in the Fig. 1(b) in the main text, where only the prior referencing spectrum is used for referencing. Figure S2 shows the actual implementation of interleaved referencing as described by Eq. 2, where the average of the prior and following reference spectrum, $\tilde{S}^{\mathrm{R}}_{n,l}(\omega)$ and $\tilde{S}^{\mathrm{R}}_{n,l+1}(\omega)$, is used as a reference spectrum, which is beneficial because this approach provides a better correction in case of

fast instrument drift. Using the same MC3T3-2 data set as above, Figure S1(c) shows that interleaved referencing suppresses interferometer drift. This suppression can be visually recognized by the absence of a linear baseline in the near-field phase spectra, $\varphi_2(\omega)$ (Fig. S1(c)) and it is further quantified by the time trace, in Fig. S1(d), blue line. Comparison with standard drift removal (Fig. S1(b)) shows that interleaved referencing (Fig. S1(c)) yields more consistent peak heights and overall shape of the near-field phase spectra. Therefore, interleaved referencing can provide the signal consistency that is required for subsequent reconstruction of IR absorption using s-SNOM models as shown in Fig. 4 in the main text.

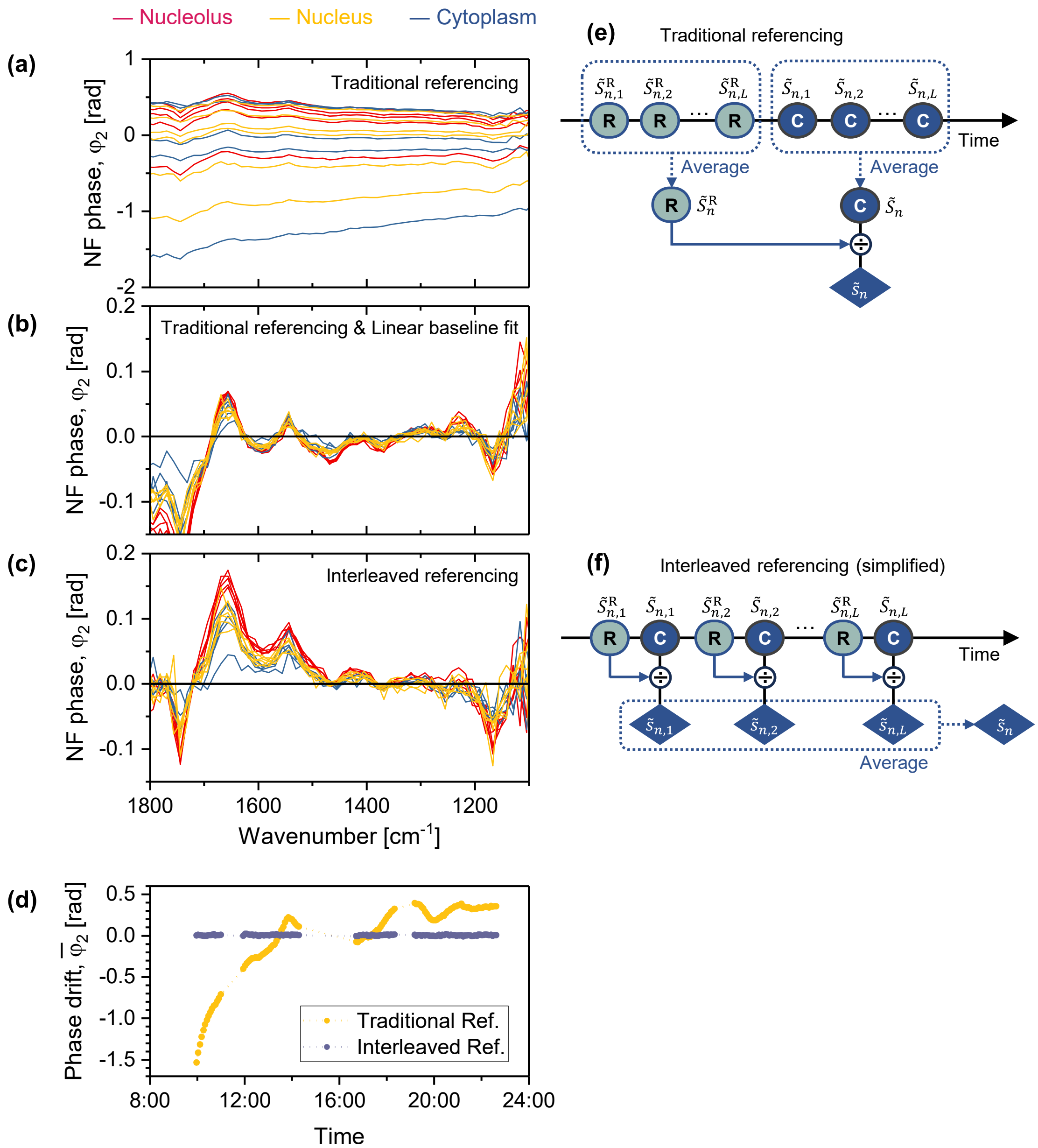

Figure S1. Effect of instrument drift in traditional vs interleaved referencing. (a) Near-field phase spectra, $\varphi_2(\omega)$, of the MC3T3-2 cell presented in the main text. Significant drift is clearly recognizable by the large linear tilt in $\varphi_2(\omega)$. (b) Standard method for drift correction by subtracting a linear fit to $\varphi_2(\omega)$ from (a) in the range between 1200 and 1700 cm^{-1}. The resulting spectra yield reduced peak height of the molecular vibrations. (c) Near-field spectra, $\varphi_2(\omega)$, as obtained with interleaved referencing. Instrument drift is eliminated, and the correct peak heights are obtained. (d) Quantification of the instrument drift of the individual measurements underlying (a) and (c) by plotting the average of the near-field phase, $\varphi_2(\omega)$, in the spectral range between 1300 and 1500 cm^{-1}, $\bar{\varphi}_n = \frac{1}{W}\sum_i \varphi_n(\omega_i)$, where W is the number of frequency points in that spectral range. (e) Schematic of traditional referencing and signal averaging of nano-FTIR spectroscopy. (f) Simplified schematic of interleaved referencing and signal averaging. R: Reference measurement on the resin adjacent to the cell, C: Measurement at a location inside the cell.

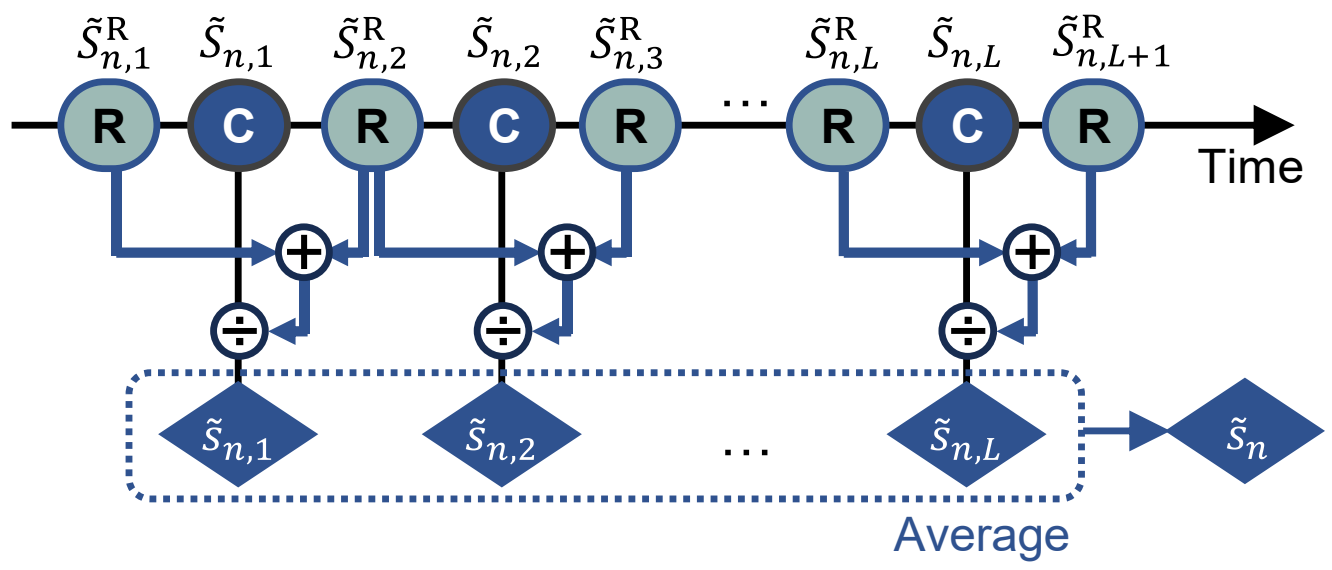

Figure S2. Schematic of the interleaved referencing for nano-FTIR spectroscopy as described by Eq. 2 in the main text, where the prior and following reference spectra are first averaged to achieve better tracking of fast instrument drift and referencing is performed using that averaged reference spectrum. R: Reference measurement on the resin adjacent to the cell, C: Measurement at a location inside the cell, $\tilde{S}_{n,l}(\omega)$: individual near-field spectrum taken at a location on the cell, $\tilde{S}^{R}_{n,l}(\omega)$: individual near-field spectrum taken on the resin, $\tilde{s}_{n,l}(\omega)$: individual referenced near-field spectrum of the location on the cell, $\tilde{s}_{n}(\omega)$: averaged referenced near-field spectrum of the location on the cell.

Supplementary Note 2: Interleaved referencing enables highly sensitive near-field phase measurements

We show that interleaved referencing can leverage long integration times to achieve a sensitivity in the near-field phase of $0.004\ \mathrm{rad}$. We also present a protocol that can be used to assess instrument noise for long nano-FTIR measurements as it is the case with the cell samples studied in the main text.

To illustrate the protocol, we use the data set of the MC3T3-2 cell presented in the main text, which in its raw data (unaveraged) form consists of a total 119 near-field spectra on the cell, $\tilde{S}_{n,l}(\omega)$, and 125 near-field spectra on the resin, $\tilde{S}^{R}_{n,l}(\omega)$. Only the reference spectra are used for this analysis as they were obtained at the same location on the sample (the resin adjacent to the cell) and thus allow us to exclusively measure instrument noise (i.e. there is no material contrast in the near-field spectra and variations in the near-field spectra can be assigned to the instrument). The same referencing protocol is applied to the data in order to obtain the same noise characteristics, in our case, this is interleaved referencing. More precisely, interleaved referencing is implemented by referencing even-numbered reference spectra, $\tilde{S}_{n,l}(\omega)$, to the odd-numbered reference spectra, $\left(\tilde{S}_{n,l-1}(\omega)+\tilde{S}_{n,l+1}(\omega)\right)/2$, as also illustrated in Fig. S3(a),

$$\tilde{s}_{n,l}(\omega)=\frac{\tilde{S}^{R}_{n,l}(\omega)}{\left[\tilde{S}^{R}_{n,l-1}(\omega)+\tilde{S}^{R}_{n,l+1}(\omega)\right]/2}, \qquad (3)$$

To increase data quality, signal averaging is performed over the referenced near-field spectra, $\tilde{s}_{n,l}(\omega)$ (Eq. (3)), as in the main text and a final referenced spectrum is obtained,

$$\tilde{s}_{n}(\omega)=s_{n}(\omega)e^{i\varphi_{n}(\omega)}=\sum_{l}\tilde{s}_{n,l}(\omega). \qquad (4)$$

Specifically, eight (8) spectra are averaged to achieve the same total measurement time as it was used in for the cell measurements presented in the main text. In case of a idealized instrument, the so-obtained

referenced near-field spectra, $\tilde{s}_n(\omega) = s_n(\omega)e^{i\varphi_n(\omega)}$, are expected to yield constant amplitude of $s_n(\omega) = 1$ and constant phase of $\varphi_n(\omega) = 0$ because all spectra were obtained at the same location on the sample (i.e., same material). In reality, the referenced spectra show significant variation. As instrument drift is already corrected, these variations are assigned to instrument noise (Fig. S3(c)). Calculating the standard deviation for every frequency ω yields a characteristic curve in the shape of a diabolo (Fig. S3(d)). The shape correlates with the available power of the illuminating source as shown by the unreferenced near-field amplitude spectra (Fig. S3(b)), yielding larger noise in the near-field phase, $\varphi_n(\omega)$, at frequency points ω, where the source power is reduced. Taking the mean value over a spectral range of significant laser power (1300 to 1700 cm^{-1}) yields an average value for the sensitivity of 0.0059 rad ($\sim 0.3°$).

We also performed this analysis for the other cell data sets (Fig. S3(e)-(g)), all of which were recorded with the nano-FTIR laser set to Band (Range) C in the microscope software. We repeated the analysis for the case that the nano-FTIR laser was set to Band B, which yields better sensitivity for the lower wavenumber end of the fingerprint spectrum (Fig. S4). The results are summarized in Table S1. The average noise level was 4.4 mrad for Band C and 3.6 mrad for Band B, which is sufficient to measure the relatively weak spectral signatures of the molecular vibrations in the cell sections with clarity.

Data Set	Band C	Band B
HeLa-1	2.8 mrad	1.6 mrad
HeLa-2	4.6 mrad	3.0 mrad
MC3T3-1	4.4 mrad	5.3 mrad
MC3T3-2	5.9 mrad	4.7 mrad

Table S1: Summary of the instrument noise of the four data sets presented in the main text. The given figures were obtained by averaging the standard deviation of the individual near-field phase spectra, $\varphi_2(\omega)$, from Figs. S3 and S4 in the spectral region of 1300 to 1700 cm^{-1} (nano-FTIR laser tuned to band C) and 1000 to 1400 cm^{-1} (nano-FTIR laser tuned to band B). Standard deviation figures are for a measurement time of about 40 min per spectrum.

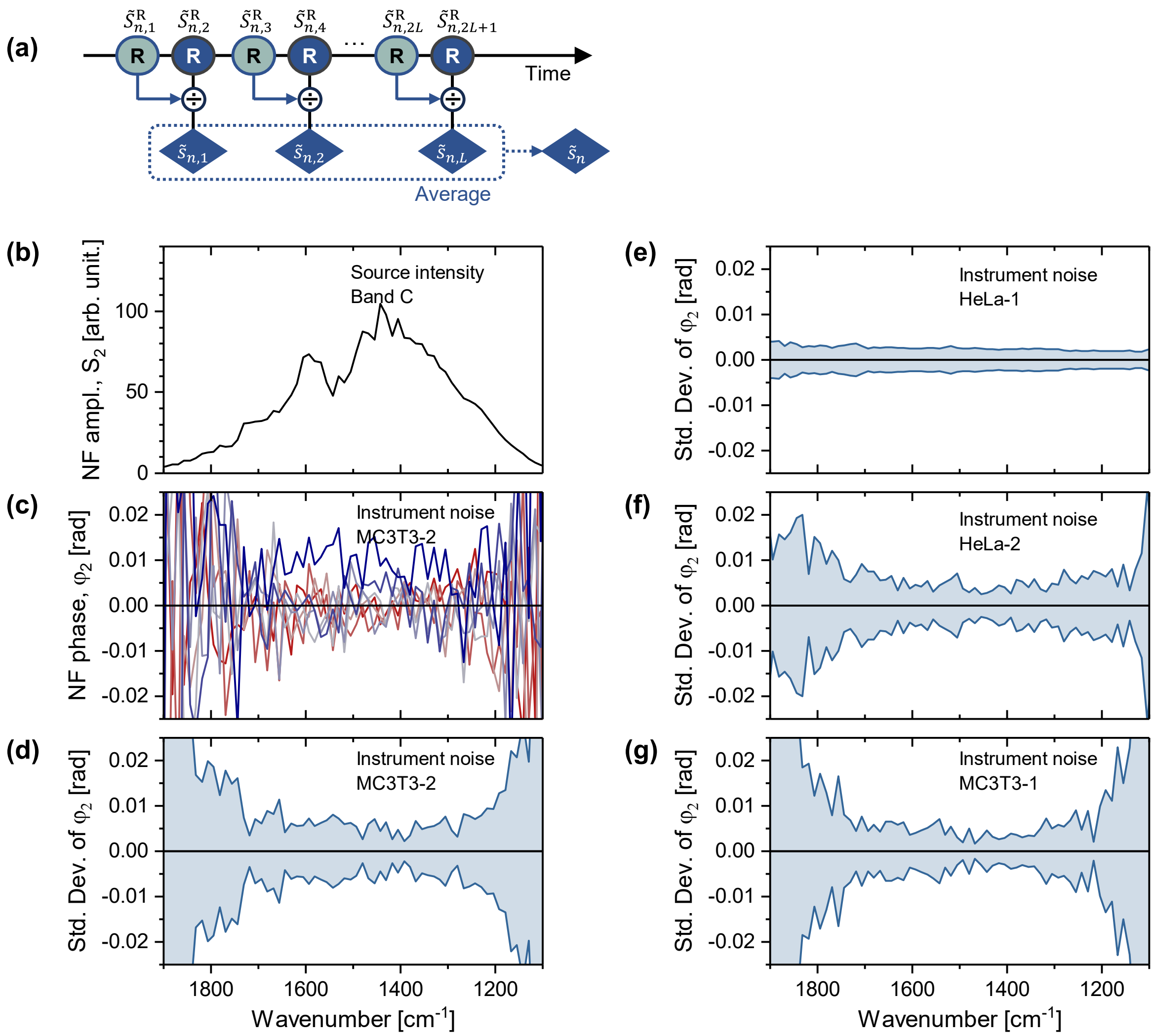

Figure S3. Characterization of instrument noise in the near-field phase. The nano-FTIR laser was configured for the **Band C** in the microscope software. (a) Measurement protocol for determining the sensitivity of nano-FTIR spectroscopy based on interleaved referencing. All measurements 'R' were done on the same location of the sample, here this was the resin adjacent to the MC3T3-2 cell. (b) Unreferenced near-field amplitude spectrum taken on the resin adjacent to the cell, showing which is indicative of the nano-FTIR laser source intensity spectrum. (c) Individual near-field phase spectra, $\varphi_2(\omega)$, as obtained with the protocol in (a). The total measurement time of each spectrum was about 40 min. (d) Standard deviation calculated over the individual spectra in (c), providing an estimate for the instrument noise of the MC3T3-2 data set. (e-g) Same as (d) but with the HeLa-1, HeLa-2 and MC3T3-1 data set.

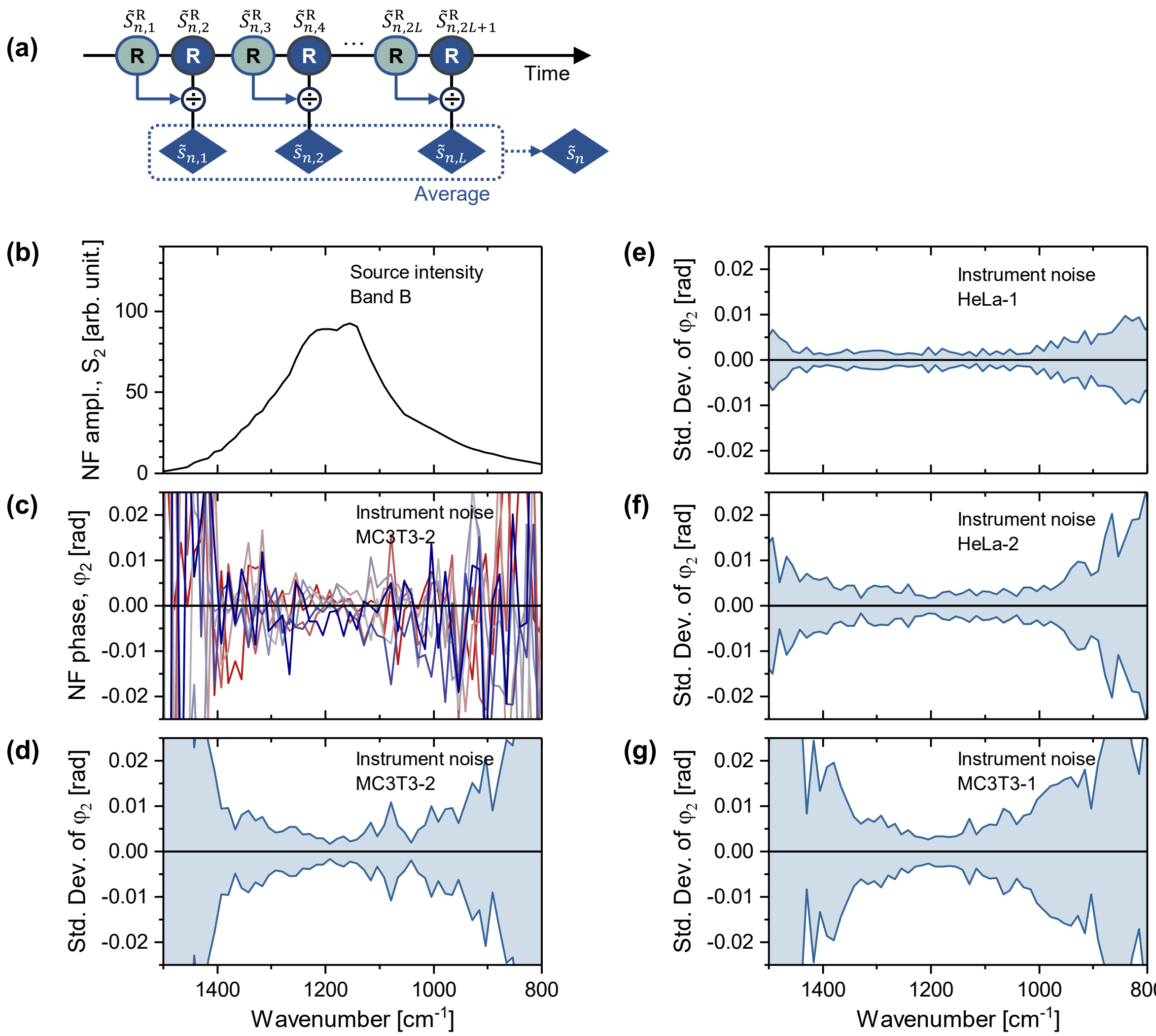

Figure S4. Characterization of instrument noise in the near-field phase. The nano-FTIR laser was configured for the **Band B** in the microscope software. (a) Measurement protocol for determining the sensitivity of nano-FTIR spectroscopy based on interleaved referencing. All measurements 'R' were done on the same location of the sample, here this was the resin adjacent to the MC3T3-2 cell. (b) Unreferenced near-field amplitude spectrum taken on the resin adjacent to the cell, showing which is indicative of the nano-FTIR laser source intensity spectrum. (c) Individual near-field phase spectra, $\varphi_2(\omega)$, as obtained with the protocol in (a). The total measurement time of each spectrum was about 40 min. (d) Standard deviation calculated over the individual spectra in (c), providing an estimate for the instrument noise of the MC3T3-2 data set. (e-g) Same as (d) but with the HeLa-1, HeLa-2 and MC3T3-1 data set.

Supplementary Note 3: Near-field images and spectra of all four cells

Figure S5 shows an optical microscopy image of the cervical cancer cell (HeLa) section, as obtained by ultramicrotome sectioning. The individual cells can be recognized by their slightly different color (lighter blue) compared to the pure resin film (darker blue) and the general features are already spatially resolved in the optical microscopy image, which helped finding the cells in near-field microscopy.

Figures S6-S9 summarize the near-field data obtained with the four cells considered in the main text. Panel (a) illustrates the topography and near-field amplitude and phase images. The locations (ROIs) on the cells where the spectroscopy was performed are marked. Note that the ROIs were selected with care to minimize the effect from inhomogeneities within the cell: (i) nucleolus: only the bright areas of the nucleolus were selected, avoiding the darker dots, (ii) nucleus and cytoplasm: homogeneous areas were chosen avoiding the occasional holes. To this end, the near-field phase image was taken as a reference and the topography mage – revealing certain features -- was used as a guide during nano-FTIR spectroscopy. Panels (b) and (c) show the raw near-field spectra obtained at the individual ranges of the DFG laser before stitching was applied. The spread among the individual near-field spectra belonging to the same subcellular region is small, which highlights the excellent repeatability of interleaved referencing for nano-FTIR spectroscopy.

Figure S10 shows the near-field phase spectra, $\varphi_2(\omega)$, and the normalized near-field phase spectra, $a_2(\omega)$, of the four considered cells in the main text.

Figure S11 shows the effect or IR absorption of the resin material on the contrast of near-field images taken at two exemplary frequencies: 1744 cm-1, where IR absorption in the resin is large and IR absorption in the cell is weak, 1660 cm-1, where IR absorption in the resin is weak and IR absorption in the cell is large.

In Figure S12, we evaluate the spatial resolution of our s-SNOM setup using a high-resolution scan across the boundary between cell and the surrounding resin. To this end, we fit an error function to the edge profile and derive the boundary width from its derivative, the Line Spread Function [1]. This analysis yields an approximation for the spatial resolution of 48 nm, which aligns well with values reported in previous s-SNOM studies of cell sections [2,3]. In comparison to conventional (far-field) IR microspectroscopy, s-SNOM thus offers a roughly 60-times improved spatial resolution (considering $\lambda/2$ as the spatial resolution of the conventional spectroscopy).

Table S2 displays the standard deviation over the near-field spectra of all ROIs of the same subcellular region and in a spectral range where laser power is highest (1000 to 1700 cm-1). The average value over all values in Table S2 is $6\ \mathrm{mrad}$. This value is slightly larger than the instrument noise (Table S1), which we attribute to small chemical differences between the ROIs of the same subcellular type.

Table S3 identifies the peak positions of the cellular IR absorption bands in the cellular absorption index spectra, $k_{\mathrm{cell}}(\omega)$, as shown in the main text.

Table S4 compares the peak positions of near-field phase spectra, $\varphi_2(\omega)$, and in the cellular absorption index spectra, $k_{\mathrm{cell}}(\omega)$, as shown in Figs. 3 and 5 in the main text.

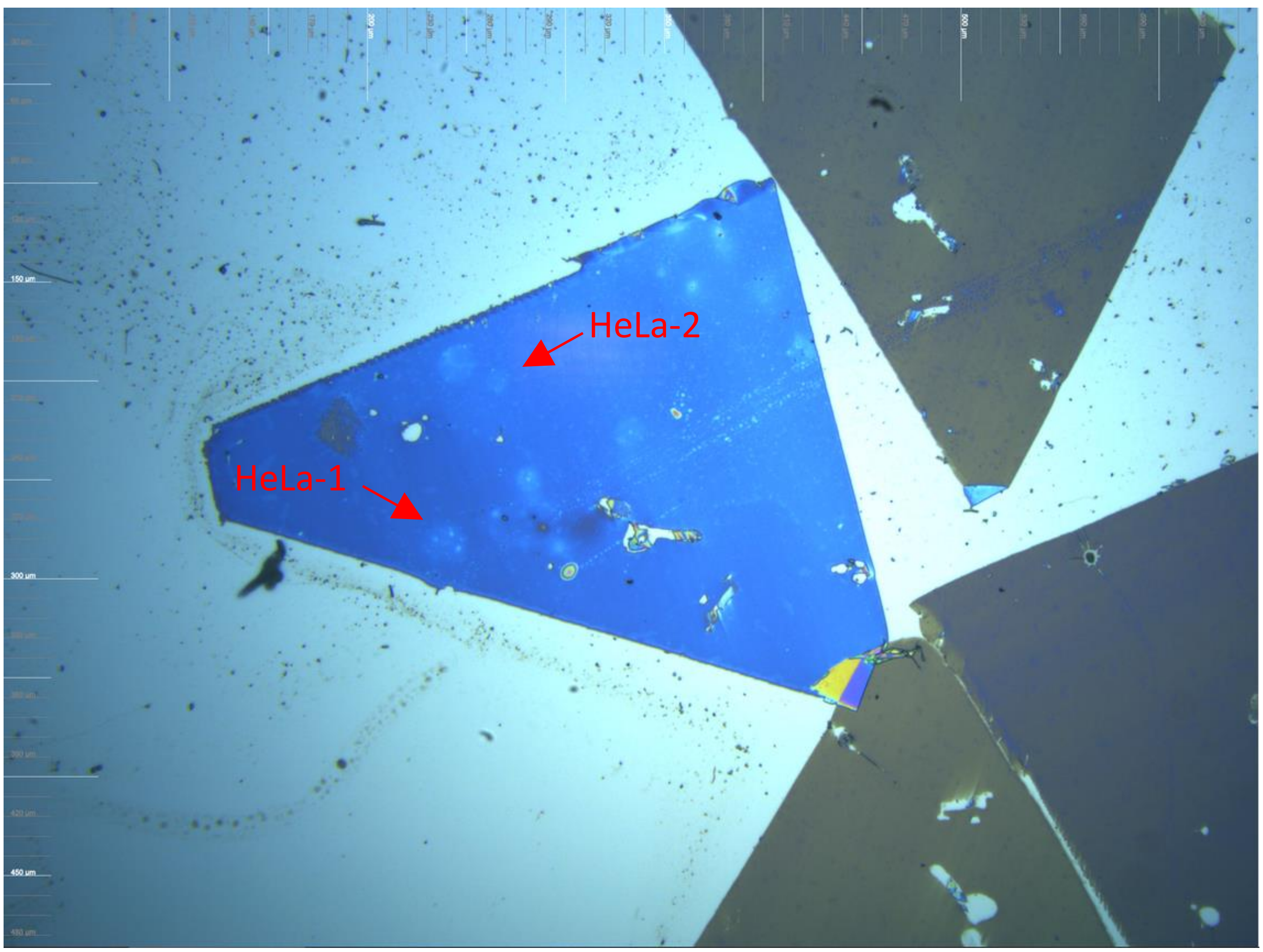

Figure S5: Optical microscopy image of the cervical cancer cell (HeLa) section. The cells HeLa-1 and HeLa-2 considered in the main text are highlighted.

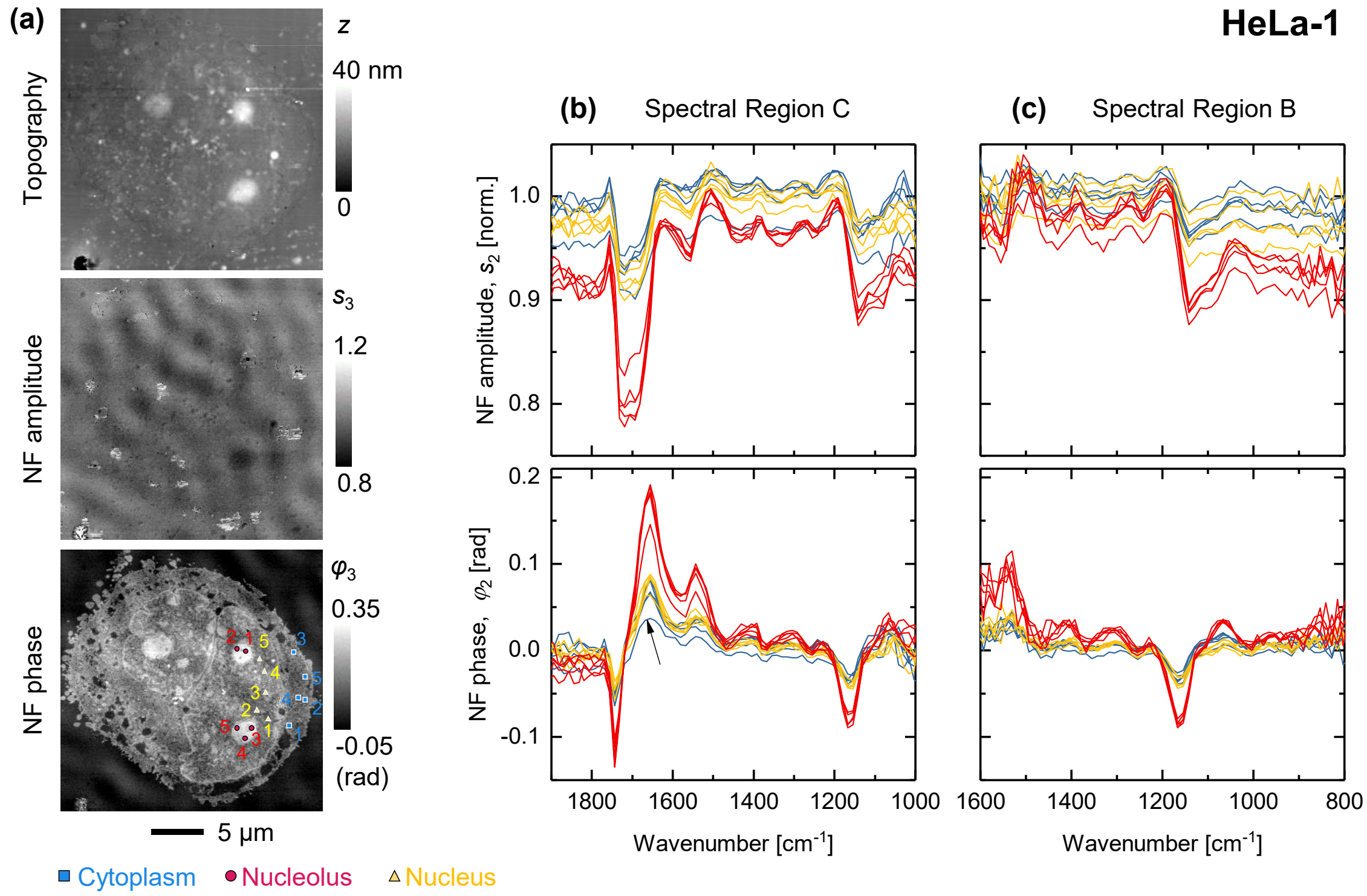

Figure S6: (a) AFM topography image, near-field amplitude, s_3, and near-field phase image, φ_3, taken at the Amide I band (1660 cm^{-1}) of the cervical cancer cell, HeLa-1. (b,c) Near-field amplitude and phase spectra, $s_2(\omega)$ and $\varphi_2(\omega)$, taken at the locations indicated in the near-field phase image in (a) and referenced to the near-field signal on the pure resin next to the cell. To this end, the DFG laser was tuned to the B band (covering a spectral range between 800 cm^{-1} and 1600 cm^{-1}) and C band (covering a spectral range between 1000 cm^{-1} and 1900 cm^{-1}). The arrow in (b), bottom indicates a spectrum that was removed manually due to low signal.

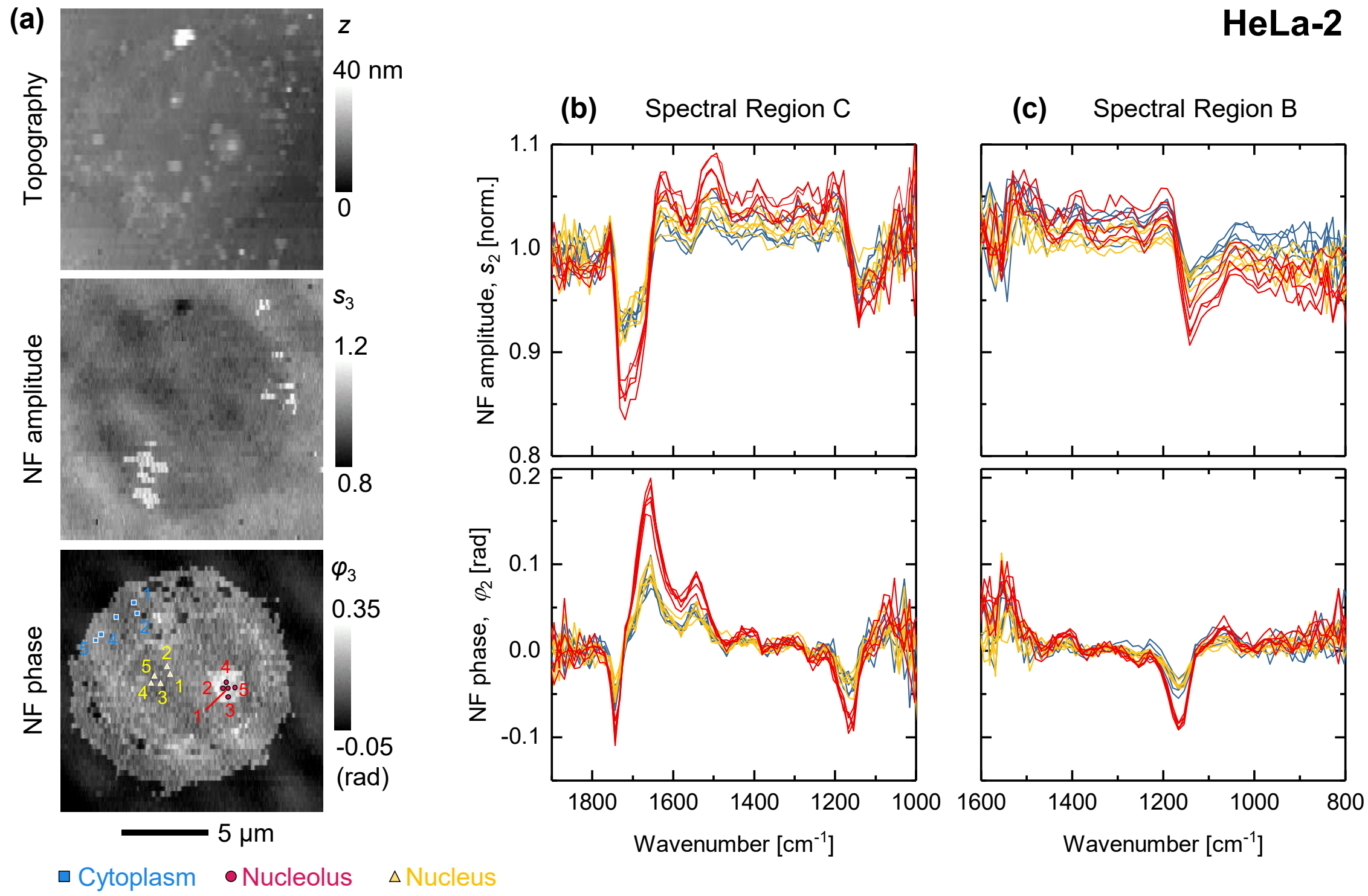

Figure S7: (a) AFM topography image, near-field amplitude, s_3, and near-field phase image, φ_3, taken at the Amide I band (1660 cm^{-1}) of the cervical cancer cell, HeLa-2. (b,c) Near-field amplitude and phase spectra, $s_2(\omega)$ and $\varphi_2(\omega)$, taken at the locations indicated in the near-field phase image in (a) and referenced to the near-field signal on the pure resin next to the cell. To this end, the DFG laser was tuned to the B band (covering a spectral range between 800 cm^{-1} and 1600 cm^{-1}) and C band (covering a spectral range between 1000 cm^{-1} and 1900 cm^{-1}).

MC3T3-1

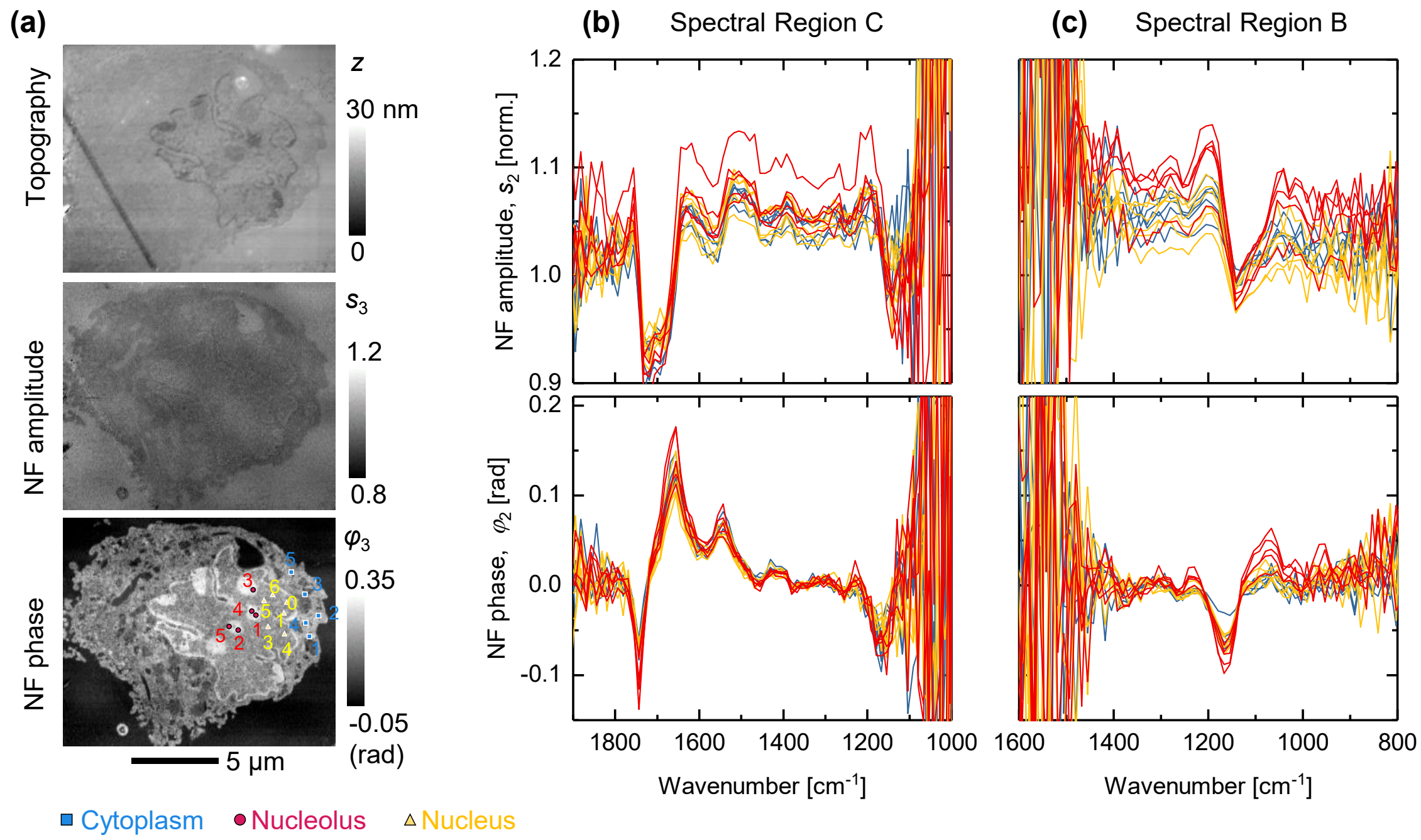

Figure S8: (a) AFM topography image, near-field amplitude, s_3, and near-field phase image, φ_3, taken at the Amide I band (1660 cm^{-1}) of the mouse osteoblast precursor cell, MC3T3-1. (b,c) Near-field amplitude and phase spectra, $s_2(\omega)$ and $\varphi_2(\omega)$, taken at the locations indicated in the near-field phase image in (a) and referenced to the near-field signal on the pure resin next to the cell. To this end, the DFG laser was tuned to the B band (covering a spectral range between 800 cm^{-1} and 1600 cm^{-1}) and C band (covering a spectral range between 1000 cm^{-1} and 1900 cm^{-1}).

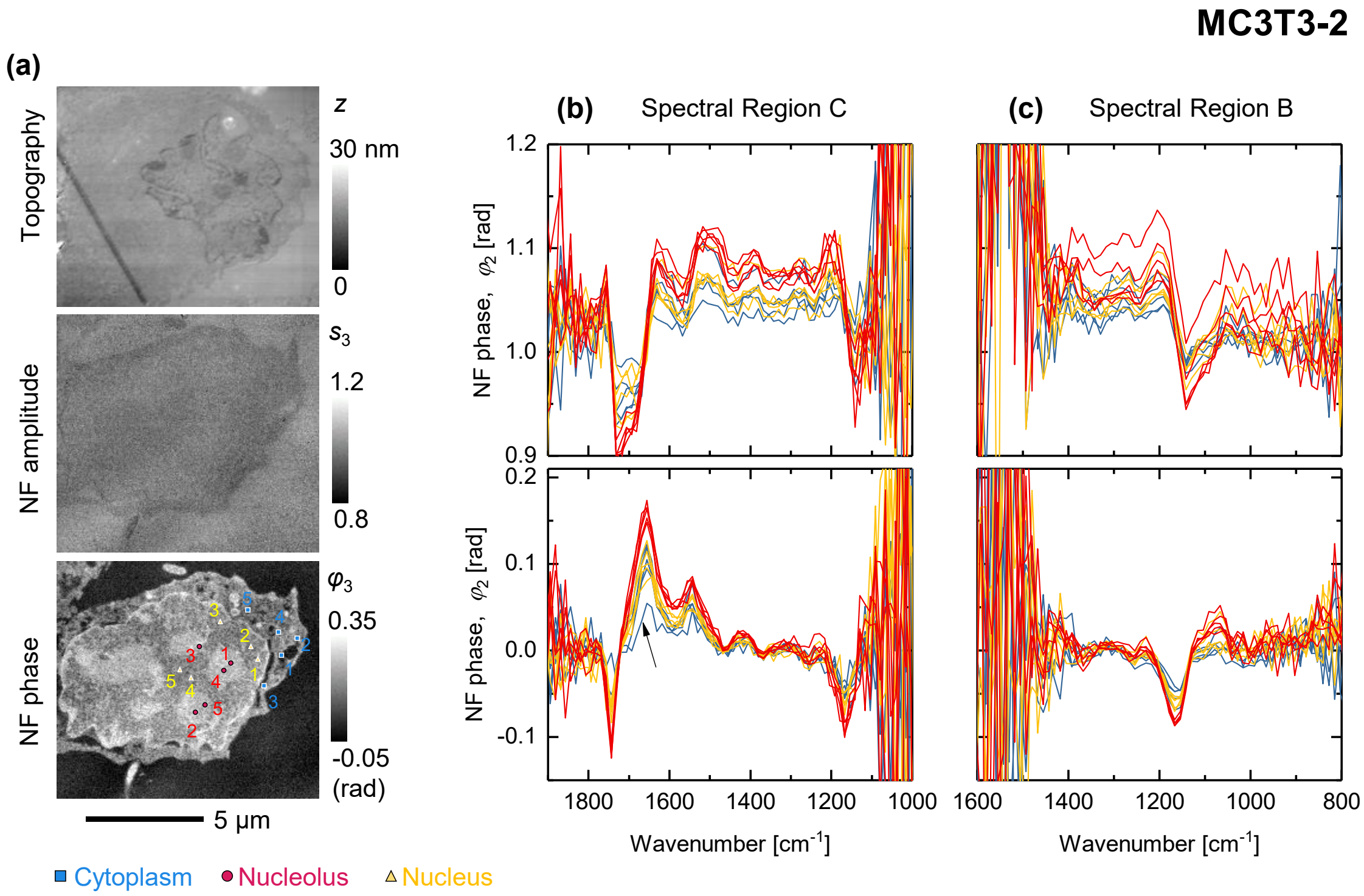

Figure S9: (a) AFM topography image, near-field amplitude, s_3, and near-field phase image, φ_3, taken at the Amide I band (1660 cm^{-1}) of the mouse osteoblast precursor cell, MC3T3-2. (b,c) Near-field amplitude and phase spectra, $s_2(\omega)$ and $\varphi_2(\omega)$, taken at the locations indicated in the near-field phase image in (a) and referenced to the near-field signal on the pure resin next to the cell. To this end, the DFG laser was tuned to the B band (covering a spectral range between 800 cm^{-1} and 1600 cm^{-1}) and C band (covering a spectral range between 1000 cm^{-1} and 1900 cm^{-1}). The arrow in (b), bottom indicates a spectrum that was removed manually due to low signal.

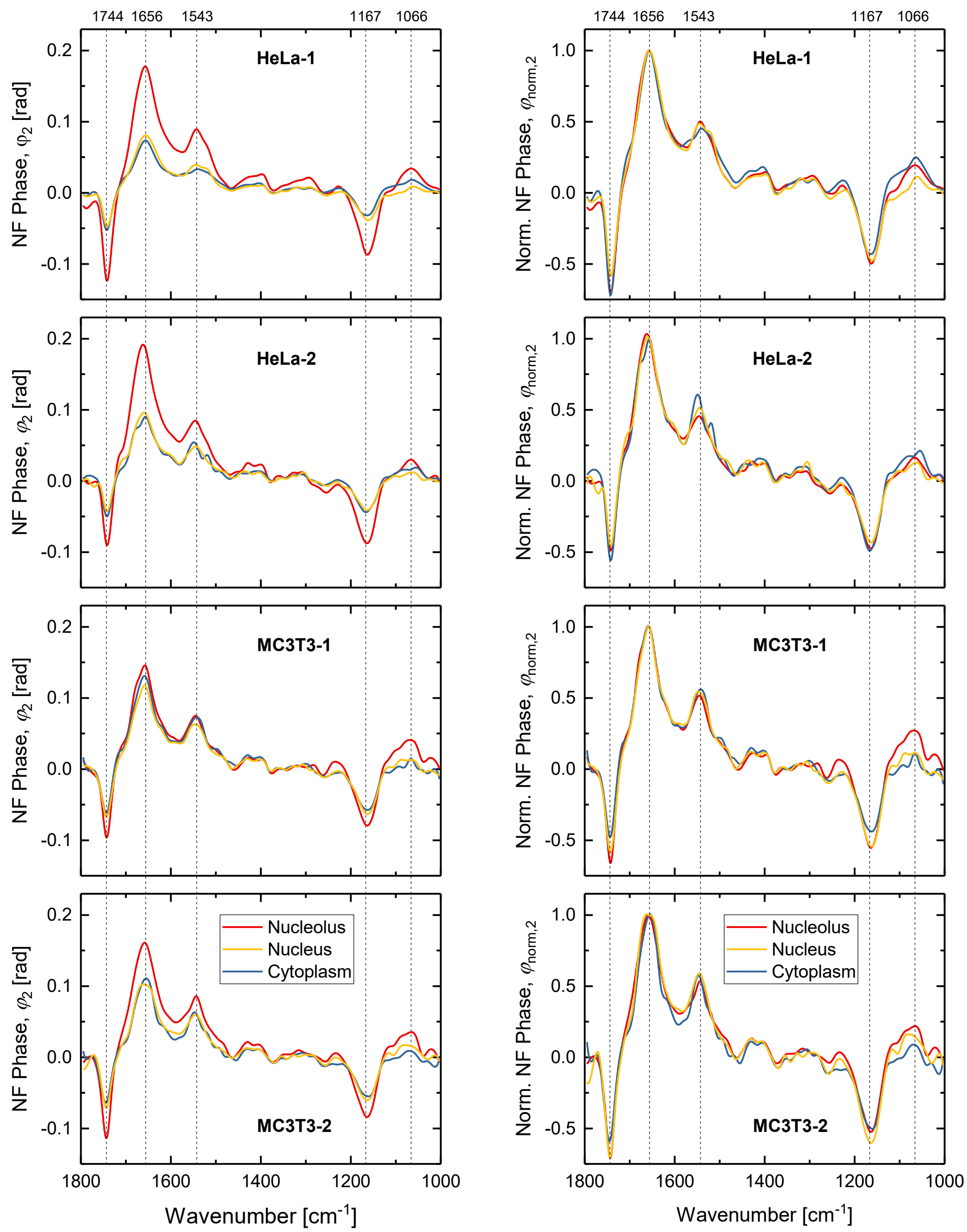

Figure S10: Left column: Near-field phase spectra, $\varphi_2(\omega)$, from the four considered cells. Right Column: Normalized near-field phase spectra, $\varphi_{\mathrm{norm},2}(\omega)$, to the Amide 1 peak at 1656 cm^{-1}.

Data Set	Cytoplasm	Nucleus	Nucleolus
HeLa-1	3.6 mrad	4.0 mrad	6.4 mrad
HeLa-2	5.7 mrad	5.3 mrad	5.5 mrad
MC3T3-1	6.2 mrad	6.5 mrad	8.5 mrad
MC3T3-2	6.4 mrad	6.9 mrad	5.8 mrad

Table S2: The standard deviation of the individual near-field phase spectra, $\varphi_2(\omega)$, in the spectral region of 1000 to 1700 cm^{-1} for three considered subcellular regions and for the for considered cells.

Band	HeLa-1	HeLa-2	MC3T3-1	MC3T3-2
Amide I	1650	1656	1654	1650
Amide II	1541	1544	1543	1543
$\delta CH_2/CH_3$ of proteins	1455	1454	1457	1455
symmetric stretching vibrations of the COO^- group of fatty and amino acids and OH deformations of ribose ring in DNA/RNA	1405	1405	1404	1406
$\nu_{\mathrm{as}} PO_2^-$ (asymmetric phosphate stretching vibration)	1244	1242	1243	1242
$\nu_{\mathrm{s}} PO_2^-$ band (symmetric phosphate stretching vibration) and C–O stretch of the ribose moiety	1065	1068	1069	1072

Table S3: Peak positions and proposed assignments for the HeLa and MC3T3 cells, as obtained by calculating the median of the peak positions observed in the reconstructed cellular absorption index spectra, $\kappa_{\mathrm{cell}}(\omega)$.

Band	Cell	Peak position in near-field phase spectra, $\varphi_n(\omega)$	Peak position in the absorption index spectra, $\kappa_{\text{cell}}(\omega)$	Peak Shift
Amide I	HeLa-1	1656	1650	-6
	HeLa-2	1660	1656	-4
	MC3T3-1	1658	1654	-4
	MC3T3-2	1658	1650	-8
Amide II	HeLa-1	1543	1541	-2
	HeLa-2	1546	1544	-2
	MC3T3-1	1545	1543	-2
	MC3T3-2	1545	1543	-2
$\nu_s PO_2^-$ band, C–O stretch	HeLa-1	1064	1065	+1
	HeLa-2	1063	1068	+5
	MC3T3-1	1066	1069	+3
	MC3T3-2	1070	1072	+2

Table S4: Comparison of peak positions in the measured near-field phase spectra, $\varphi_2(\omega)$, and the reconstructed cellular absorption index spectra, $\kappa_{\text{cell}}(\omega)$, as obtained by determining the position of the peak maximum in the spectra in Figs. 3, 5 in the main text.

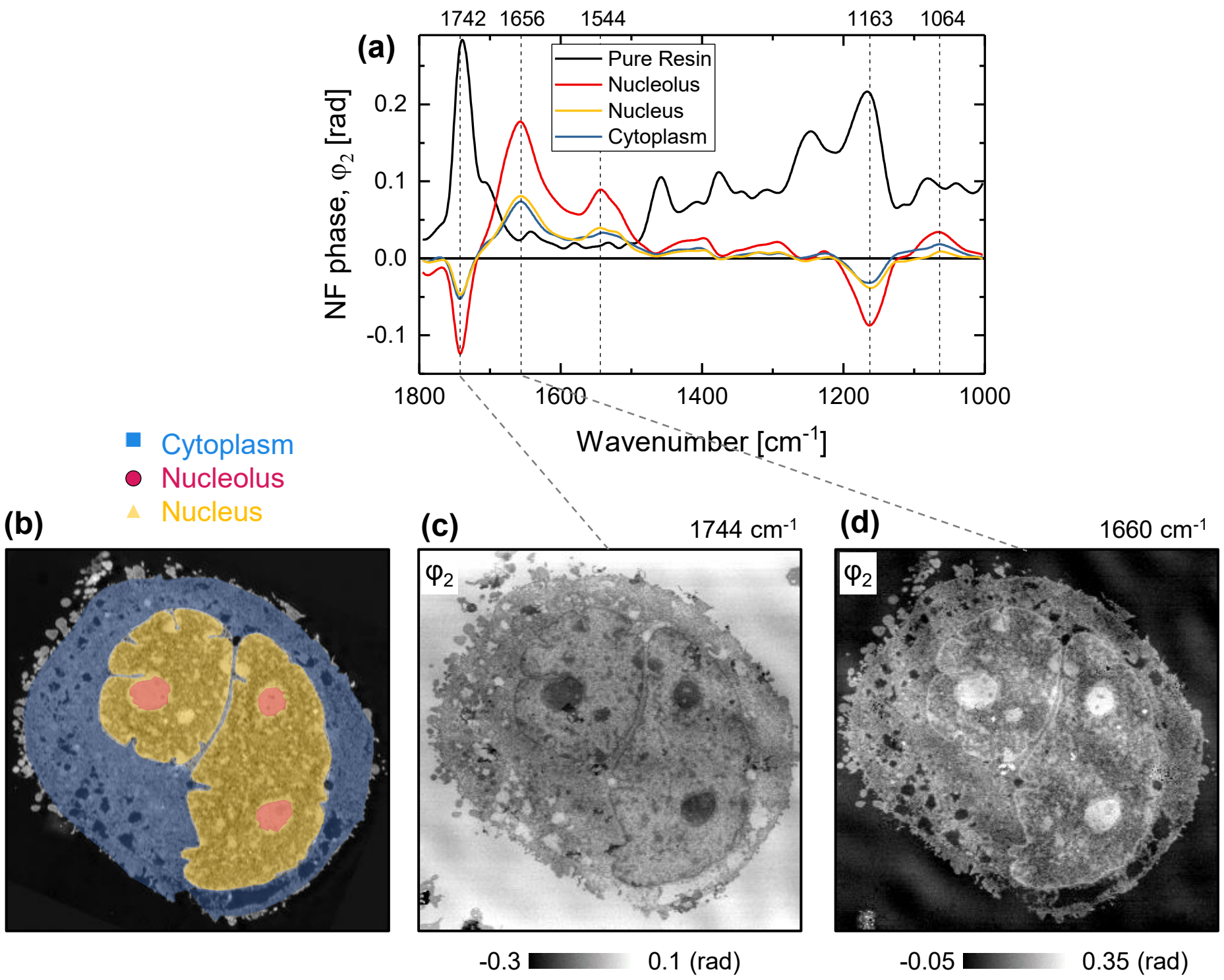

Figure S11: Near-field phase contrast inversion due to IR absorption in the embedding resin. (a) Referenced near-field phase spectra of the three considered subcellular regions, $\varphi_3(\omega)$, from Fig. 2(e) from the main text. (b) SEM image of a single HeLa cell section. Cytoplasm, nucleolus and nucleus regions are colored coded as shown in the legend above. (c) Near-field phase image measured at the resin absorption line at 1744 cm^{-1}. (d) Near-field phase image measured at the Amide I band at 1660 cm^{-1}. The contrast in the images (c),(d) corresponds to the peak intensity at 1744 cm^{-1} and 1660 cm^{-1} in the spectra in (a). When IR absorption of the cell material is prevailing (1660 cm^{-1}), the higher material concentration of nucleolus (red) yields a larger peak in the spectrum (a) and larger phase signal in the image (d). Conversely, when IR absorption in the resin is prevailing (1744 cm^{-1}), the higher material concentration of nucleolus (red) results in a less resin concentration and a deeper dip in the spectrum (a) and more negative phase signal in the image (c).

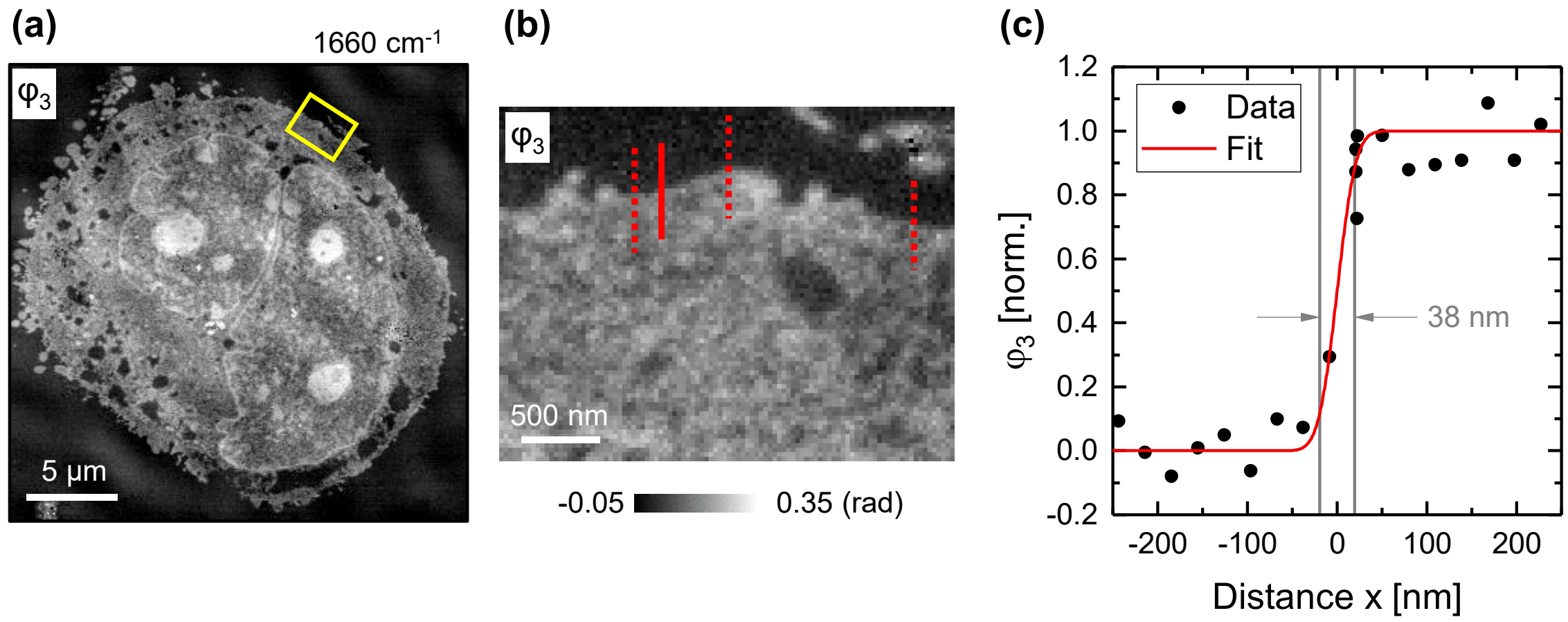

Figure S12: Determination of the spatial resolution with the HeLa-1 cell image. (a) Near-field phase image, φ_3, taken at 1660 cm^{-1} wavenumber from Fig. 2(b) from the main text. (b) High-resolution near-field image taken at the region marked by the fellow box in (a). (c) Line profile taken along the solid red line in (b) at the edge of the cell section (dots). The red line marks the fit of the error function to the data. The width of the edge was determined as 38 nm using the Line Spread Function. The average edge width taken over four randomly selected locations in (b) is $48\ \mathrm{nm} \pm 14\ \mathrm{nm}$.

References

1. S. Mastel, A. A. Govyadinov, C. Maissen, A. Chuvilin, A. Berger, and R. Hillenbrand, "Understanding the Image Contrast of Material Boundaries in IR Nanoscopy Reaching 5 nm Spatial Resolution," ACS Photonics **5**, 3372–3378 (2018).
2. K. Kanevche, D. J. Burr, D. J. Nürnberg, P. K. Hass, A. Elsaesser, and J. Heberle, "Infrared nanoscopy and tomography of intracellular structures," Commun. Biol. **4**, 1–8 (2021).
3. G. E. Greaves, D. Kiryushko, H. W. Auner, A. E. Porter, and C. C. Phillips, "Label-free nanoscale mapping of intracellular organelle chemistry," Commun. Biol. **6**, 1–7 (2023).
4. S. Mastel, A. A. Govyadinov, T. V. A. G. de Oliveira, I. Amenabar, and R. Hillenbrand, "Nanoscale-resolved chemical identification of thin organic films using infrared near-field spectroscopy and standard Fourier transform infrared references," Appl. Phys. Lett. **106**, 023113 (2015).
5. A. A. Govyadinov, I. Amenabar, F. Huth, P. S. Carney, and R. Hillenbrand, "Quantitative Measurement of Local Infrared Absorption and Dielectric Function with Tip-Enhanced Near-Field Microscopy," J. Phys. Chem. Lett. **4**, 1526–1531 (2013).
